# Lack of *Tgfbr1* and *Acvr1b* synergistically stimulates myofibre hypertrophy and accelerates muscle regeneration

**DOI:** 10.7554/eLife.77610

**Published:** 2022-03-24

**Authors:** Michèle MG Hillege, Andi Shi, Ricardo A Galli, Gang Wu, Philippe Bertolino, Willem MH Hoogaars, Richard T Jaspers

**Affiliations:** 1 Laboratory for Myology, Department of Human Movement Sciences, Faculty of Behavioural and Movement Sciences, Vrije Universiteit Amsterdam, Amsterdam Movement Sciences Amsterdam Netherlands; 2 Department of Oral and Maxillofacial Surgery/Pathology, Amsterdam UMC and Academic Center for Dentistry Amsterdam (ACTA), Vrije Universiteit Amsterdam, Amsterdam Movement Sciences Amsterdam Netherlands; 3 Department of Prosthodontics, Affiliated Stomatology Hospital of Guangzhou Medical University, Guangdong Engineering Research Center of Oral Restoration and Reconstruction, Guangzhou Key Laboratory of Basic and Applied Research of Oral Regenerative Medicine Guangzhou China; 4 Department of Oral Cell Biology, Academic Centre for Dentistry Amsterdam (ACTA), University of Amsterdam and Vrije Universiteit Amsterdam, Amsterdam Movement Sciences Amsterdam Netherlands; 5 Centre de Recherche en Cancérologie de Lyon, UMR INSERM U1052/CNRS 5286, Université de Lyon, Centre Léon Bérard Lyon France; 6 Department of Biomedical Sciences of Cells and Systems, University of Groningen, University Medical Center Groningen Groningen Netherlands; University of Cambridge United Kingdom; Icahn School of Medicine at Mount Sinai United States

**Keywords:** TGF-β, myostatin, type I receptor, hypertrophy, injury, skeletal muscle, Mouse

## Abstract

In skeletal muscle, transforming growth factor-β (TGF-β) family growth factors, TGF-β1 and myostatin, are involved in atrophy and muscle wasting disorders. Simultaneous interference with their signalling pathways may improve muscle function; however, little is known about their individual and combined receptor signalling. Here, we show that inhibition of TGF-β signalling by simultaneous muscle-specific knockout of TGF-β type I receptors *Tgfbr1* and *Acvr1b* in mice, induces substantial hypertrophy, while such effect does not occur by single receptor knockout. Hypertrophy is induced by increased phosphorylation of Akt and p70S6K and reduced E3 ligases expression, while myonuclear number remains unaltered. Combined knockout of both TGF-β type I receptors increases the number of satellite cells, macrophages and improves regeneration post cardiotoxin-induced injury by stimulating myogenic differentiation. Extra cellular matrix gene expression is exclusively elevated in muscle with combined receptor knockout. *Tgfbr1* and *Acvr1b* are synergistically involved in regulation of myofibre size, regeneration, and collagen deposition.

## Introduction

Muscle wasting disorders, such as muscular dystrophies, cancer cachexia, and sarcopenia are characterised by reduced muscle mass, impaired regeneration and fibrosis, which results in progressive muscle weakness. The transforming growth factor β (TGF-β) superfamily members TGF-β1, myostatin and activin A are involved in various processes within muscle tissue and overexpression of these proteins contributes to muscle wasting pathologies (Bernasconi et al., 1995; Carlson et al., 2008; Chen et al., 2014; Costelli et al., 2008; Leger et al., 2008; Tanaka et al., 1993).

TGF-β signalling negatively affects muscle growth by both affecting satellite cells (SCs) and myofibres. TGF-β1, myostatin and activin A inhibit myoblast differentiation (Langley et al., 2002; Liu et al., 2001; Trendelenburg et al., 2012). Inhibition of myostatin and activin A synergistically results in muscle hypertrophy (Amirouche et al., 2009; Chen et al., 2017; Chen et al., 2014; Latres et al., 2017; McFarlane et al., 2006; Zimmers et al., 2002). These effects are at least partly independent of SCs (Lee et al., 2012). In addition, TGF-β1 overexpression in vivo may also cause muscle atrophy (Mendias et al., 2012; Narola et al., 2013).

Transient TGF-β1 expression may play an essential role during muscle regeneration. TGF-β1 is expressed by inflammatory cells, such as macrophages, monocytes, and neutrophils as well as by fibroblasts after acute injury (Assoian et al., 1987; Grotendorst et al., 1989; Lawrence et al., 1984; Zimowska et al., 2009). During muscle regeneration, TGF-β1 is involved in the regulation of the immune response and plays an important role in rebuilding extracellular matrix (ECM) (Gillies and Lieber, 2011; Kehrl et al., 1986; Reibman et al., 1991; Tsunawaki et al., 1988; Wahl et al., 1987; Wiseman et al., 1988). However, chronic increased expression of TGF-β is known to contribute to muscle fibrosis (Li et al., 2004). Furthermore, myostatin and activin A have also been suggested to induce substantial skeletal muscle fibrosis (Chen et al., 2014; Li et al., 2008). Thus, inhibition of TGF-β signalling in the myofibre may substantially reduce connective tissue deposition.

In animal models, such as murine X-linked muscular dystrophy (mdx) mice, a Duchenne muscular dystrophy (DMD) mouse model, cancer cachexia mouse models or aged mice, inhibiting signalling of one or more of these ligands had beneficial effects, such as reduction in fibrosis and maintenance of muscle mass (Andreetta et al., 2006; Chen et al., 2017; Greco et al., 2015; Latres et al., 2015; Murphy et al., 2011a). Lack of either TGF-β or myostatin has been suggested to improve regeneration after acute injury (Accornero et al., 2014; McCroskery et al., 2005). Therefore, inhibiting these growth factors may be a promising therapeutic strategy to alleviate muscle wasting pathologies.

However, interference with signalling of TGF-β family members may be complicated. TGF-β family members regulate various cellular processes throughout the body, thus systemic inhibition of these growth factors may have severe consequences. Furthermore, due to overlap in function of these ligands, inhibition of a single ligand is likely not effective.

Simultaneous inhibition of TGF-β1, myostatin and activin A through interference with their downstream receptors may be an effective approach. The TGF-β family consists of at least 33 cytokines that can roughly be divided into the TGF-β/myostatin/activins subgroup and the growth differentiation factor/bone morphogenetic protein (BMP) group that often have opposing effects. These cytokines regulate gene expression via specific binding to distinct type II and type I receptors. TGF-β1 mainly signals via the type II receptor, TGF-β receptor type-2 (TGFBR2), and via the type I receptor, TGF-β receptor type-1 (TGFBR1 or ALK5). Myostatin signals via the type II receptor, activin receptor type-2B (ACVR2B), and activin A signals via type II receptors, activin receptor type-2A (ACVR2A) and ACVR2B. Activin A signals via type I receptor, activin receptor type-1B (ACVR1B or ALK4). Myostatin has been shown to signal via both TGFBR1 and ACVR1B in various cell types (Kemaladewi et al., 2012; Rebbapragada et al., 2003; ten Dijke et al., 1994).

Interference with myostatin/activin A signalling by blocking their type II receptors ACVR2A/B may not be an appropriate strategy, since these receptors are also involved in BMP signalling, which stimulates muscle hypertrophy whereas myostatin/activin A signalling is associated with atrophy (Sartori et al., 2014; Tsuchida et al., 2008). Moreover, interference with signalling via these receptors may cause severe side effects, such as nose and gum bleeds, as has been shown in in DMD boys treated with soluble ACVR2B (Campbell et al., 2017). Inhibition of type I receptors TGFBR1 and ACVR1B may provide a more specific and effective approach to alleviate muscle wasting pathologies (Sartori et al., 2014; Tsuchida et al., 2008). However, very little is known about the role of these receptors in the regulation of skeletal muscle mass and regeneration.

The aim of this study was to obtain insight in how myofibre-specific knockout of type I receptors *Tgfbr1* and *Acvr1b* affected muscle size as well as early muscle regeneration, inflammation and collagen deposition in both intact and injured muscle. We hypothesised that individual knockout of these TGF-β type I receptors would have marginal effects. Moreover, simultaneous inhibition of these type I receptors would substantially increase muscle size and enhance early myofibre regeneration, while attenuating fibrosis.

## Results

### *Acvr1b* and *Tgfbr1* expression was successfully reduced after tamoxifen treatment

**Figure 1.**
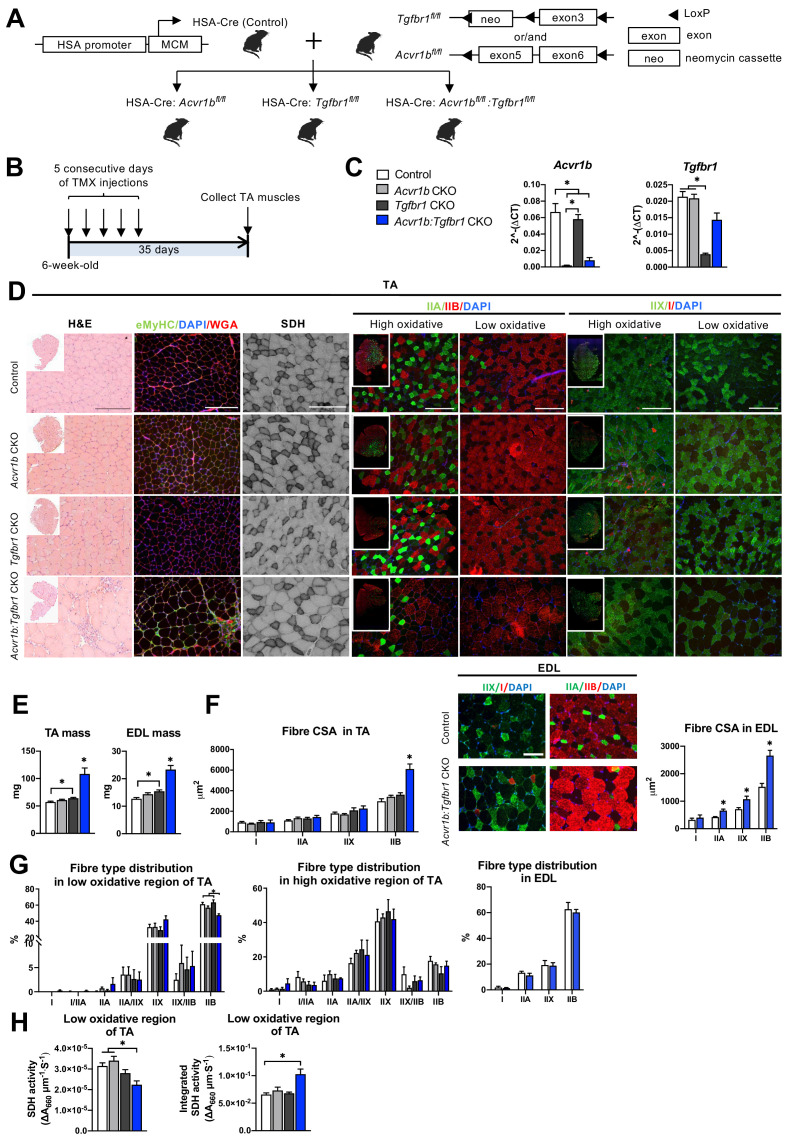
Simultaneous knockout of both *Acvr1b* and *Tgfbr1* caused muscle hypertrophy. (**A**) Scheme showing cross-breeding of HSA-Cre mouse line with conditional knockout mouse lines *Acvr1b^fl/fl^* and *Tgfbr1^fl/fl^*. LoxP sites are indicated by black arrows. A loxP-flanked neomycin (neo) cassette is inserted upstream of exon3 of *Acvr1b* genome. (**B**) Scheme demonstrating receptor knockout induced by tamoxifen (TMX) injection for consecutive 5 days. (**C**) Relative mRNA expression of *Acvr1b* and *Tgfbr1* in TA muscles of experimental groups. (**D**) Histology stainings of TA muscles 35 days after first TMX injection. H&E staining and immunofluorescent staining of eMyHC (green) of TA showed regenerative regions containing eMyHC^+^ myofibres with central nuclei (DAPI, blue) in *Acvr1b:Tgfbr1* CKO mice, wheat glucose agglutinin (WGA, red) was used to visualise cell membranes and ECM. *Acvr1b:Tgfbr1* CKO mice showed lower staining intensity for SDH activity in low oxidative region of TA. MyHCs staining demonstrated type IIA (green), IIB (red), IIX (green) and I (red) myofibres in low and high oxidative regions of TA. Scale bars = 250 μm. (**E**) TA and EDL muscle mass and myofibre cross-sectional areas (CSAs) were increased in *Acvr1b:Tgfbr1* CKO mice. (**F**) In TA, specifically CSA of type IIB myofibres was increased in *Acvr1b:Tgfbr1* CKO animals, while in EDL CSA of all type II myofibres was increased. Myofibre types were stained in EDL. (**G**) Percentage of type IIB in low oxidative region of TA was reduced. No differences were observed in myofibre distribution in high oxidative region of TA or EDL. (**H**) SDH activity (absorbance units (∆A_660_) per micrometer section thickness per second of incubation time (∆A_660_∙μm^–1^∙s^–1^)) was decreased, while the integrated SDH activity, SDH activity multiplied by CSA (∆A_660_∙μm∙s^–1^), increased in low oxidative region of TA of *Acvr1b:Tgfbr1* CKO animals. N = 5–8 mice. Results are presented as mean + SEM. *: p < 0.05. Significant difference between individual groups is indicated by lines with a *. Single * indicates significant difference compared to all other groups. Figure 1—source data 1.Quantification of Acvr1b and Tgfbr1 gene expression levels in TA and myofiber phenotype in TA and EDL in absence of injury.

The aim of this study was to investigate effects of mature myofibre-specific knockout of *Tgfbr1* and *Acvr1b* on muscle morphology as well as early muscle regeneration, inflammation and collagen deposition in both uninjured muscle tissue and after acute cardiotoxin (CTX) injury. For this purpose, the HSA-Cre mouse line (McCarthy et al., 2012b), that expresses tamoxifen (TMX) inducible Cre under a human α-skeletal actin (*ACTA1*) promotor (HSA) was cross bred with the conditional knockout of *Acvr1b^fl/fl^* (Ripoche et al., 2013) or *Tgfbr1^fl/fl^* (Larsson et al., 2001) mouse lines to obtain mouse lines HSA-Cre:*Acvr1b^fl/fl^*, HSA-Cre:*Tgfbr1^fl/fl^*, and HSA*-*Cre*:Acvr1b ^fl/fl^:Tgfbr1 ^fl/fl^* (further referred to as *Acvr1b* CKO*, Tgfbr1* CKO and *Acvr1b:Tgfbr1* CKO). Receptors were deleted when mice were 6 weeks old (Figure 1A and B).

Expression levels of *Acvr1b* and *Tgfbr1* mRNA showed successful knockout as *Acvr1b* mRNA levels in tibialis anterior (TA) muscles were reduced in *Acvr1b* CKO animals by 97% and in *Acvr1b:Tgfbr1* CKO animals by 88%. *Tgfbr1* expression levels in TA muscles were reduced in *Tgfbr1* CKO animals by 82%. Unexpectedly, *Tgfbr1* expression levels in TA muscle of *Acvr1b:Tgfbr1* CKO animals were not significantly reduced compared to those of control animals (Figure 1C). Note, however, that lack of significantly reduced *Tgfbr1* expression is likely a consequence of high *Tgfbr1* expression by other cell types present within the muscle, rather than of unsuccessful knockdown. This issue is addressed below in more detail (see Figure 3). *Acvr1b* expression levels did not affect *Tgfbr1* expression levels and vice versa.

### Simultaneous knockout of *Acvr1b* and *Tgfbr1* resulted in type IIB myofibre hypertrophy and had modest effects on myofibre type distribution

TGFBR1 and ACVR1B ligands are well known for their regulatory effects on muscle mass. Here, TA mass of *Acvr1b:Tgfbr1* CKO mice (108.4 ± 11.0 mg) was nearly doubled compared to that of control animals (57.2 ± 1.5 mg). TA mass of *Tgfbr1* CKO mice (64.2 ± 1.4 mg) was also increased, however to a much lower extend. TA mass of *Acvr1b* CKO mice (60.8 ± 1.5 mg) did not differ from that of controls (Figure 1E). To test whether the observed effects in TA also applied to other muscles, extensor digitorum longus muscle (EDL) mass was determined. Similar to TA muscle, EDL mass of *Acvr1b:Tgfbr1* CKO (23.3 ± 1.5 mg) and *Tgfbr1* CKO mice (15.4 ± 0.5 mg) was increased by 1.8-fold and 1.3-fold, compared to that of control mice (12.7 ± 0.4 mg), respectively, while EDL mass of *Acvr1b* CKO mice (14.4 ± 0.5 mg) did not differ from that of control mice (Figure 1E). Note that in TA, specifically the cross-sectional area (CSA) of type IIB myofibres of *Acvr1b:Tgfbr1* CKO mice was twofold larger compared to that of control animals (Figure 1F), indicating that simultaneous knockout of both *Acvr1b* and *Tgfbr1* synergistically causes myofibre hypertrophy in type IIB myofibres. In contrast to observations in TA, CSA of type IIA and type IIX myofibres in EDL muscle of *Acvr1b:Tgfbr1* CKO mice were increased by 1.6-fold and 1.5-fold compared to those of control mice, respectively. However, similar to TA muscle, CSA of type IIB myofibres of *Acvr1b:Tgfbr1* CKO mice was increased most substantially compared to that of control mice (1.7-fold) (Figure 1F).

These results indicate that simultaneous knockout of *Acvr1b* and *Tgfbr1* in mature mouse myofibre synergistically causes muscle hypertrophy of mostly type IIB myofibres, whereas individual knockout has little effect on muscle mass or myofibre CSA.

Observed effects on myofibre CSA may indicate alterations in myofibre metabolism as well as in myofibre type distribution. Therefore, myofibre type distribution was determined in both the high and low oxidative region of the TA (Figure 1D and G). In the high oxidative region no significant differences were observed. However, in the low oxidative region of *Acvr1b:Tgfbr1* CKO animals the percentage of type IIB myofibres was lower (47% ± 2%) compared to both control (61% ± 2%) and *Tgfbr1* CKO animals (63% ± 3%) (Figure 1G), which indicates a shift toward an oxidative phenotype in these muscles. In contrast, no differences in EDL myofibre type distribution were observed between *Acvr1b:Tgfbr1* CKO and control mice (Figure 1F and G).

Taken together, lack of both *Acvr1b* and *Tgfbr1* reduces the percentage of type IIB myofibres in TA muscle, but has only modest effects on myofibre type distribution.

### Type IIB myofibre hypertrophy resulted in reduced SDH activity

In skeletal muscle, myofibre size and oxidative capacity are inversely related, indicating that metabolism implies a size constraint (Van Der Laarse et al., 1997; van Wessel et al., 2010). To test whether the excessive hypertrophy within the low oxidative region of the TA muscles was accompanied by a reduction in oxidative metabolism, succinate dehydrogenase (SDH) activity and integrated SDH activity were determined. In *Acvr1b:Tgfbr1* CKO mice SDH activity was decreased by 30% compared to that in *Acvr1b* CKO and control animals. However, the integrated SDH activity (total oxidative capacity of myofibres) in *Acvr1b:Tgfbr1* CKO mice was increased by 60% compared to that in control animals. This suggests that while locally the total oxidative capacity in the low oxidative region of TA muscle of *Acvr1b:Tgfbr1* CKO animals may be reduced, the oxidative capacity per myofibre in the low oxidative region of TA muscle of these animals was substantially increased (Figure 1H).

### Myofibres with central nuclei and increased number of SCs were observed in TA muscle of *Acvr1b:Tgfbr1* CKO mice

**Figure 2.**
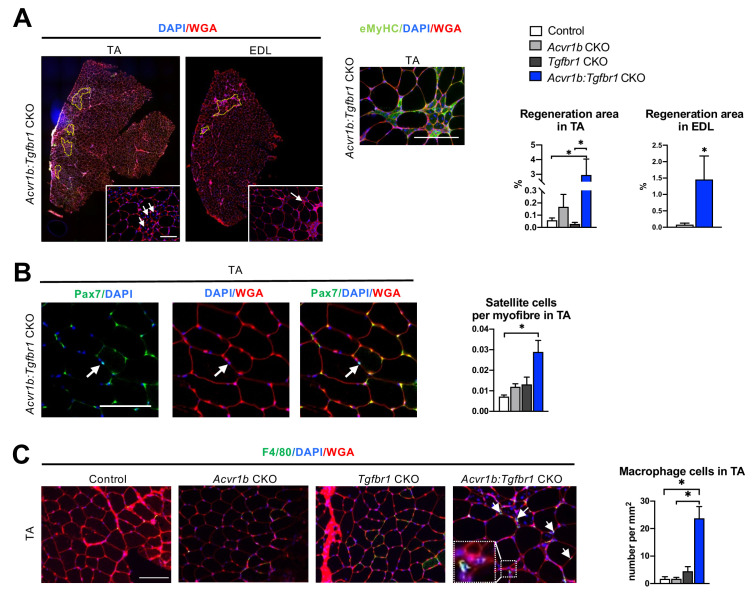
Increased heterogeneity of cell types was found in both TA and EDL of *Acvr1b:Tgfbr1* CKO animals. (**A**) Regions with spontaneously regenerating myofibres (circled by yellow dash lines) with central nuclei (indicated by arrows) were particularly present in low oxidative region of TA and EDL of *Acvr1b:Tgfbr1* CKO animals. (**B**) Increased number of Pax7^+^ cells per myofibre was found in TA of *Acvr1b:Tgfbr1* CKO mice. (**C**) IF staining of F4/80 (green) showed an increased number of macrophages (indicated by arrows) in TA muscle per mm^2^ CSA of *Acvr1b:Tgfbr1* CKO mice compared to control. Macrophages (image with higher magnification on the left corner) were mainly located around myofibres with central nuclei. Scale bar = 100 µm. N = 5–8 mice. Results are presented as mean + SEM. *. p < 0.05. Significant differences between individual groups are indicated by lines with a *. Single * indicates significant difference compared to all other groups at the same time point. Figure 2—source data 1.Quantification of number of regenerating myofibres, satellite cells and macrophages in absence of injury.

Hematoxylin & Eosin (H&E) staining and embryonic myosin heavy chain (eMyHC) staining showed within TA of uninjured *Acvr1b:Tgfbr1* CKO animals, regions with small myofibres with centrally located nuclei, indicating injured myofibres. These myofibres were eMyHC^+^ and surrounded by many other cells, likely a combination of SCs, fibroblasts and immune cells (Figure 1D). These regions with regenerating myofibres were mainly present in the low oxidative region of the TA and comprised on average 2.95% of the muscle CSA. These regions were almost never observed in TA of other animals ( < 0.2%) (Figure 2A). Similar regions with myofibres containing centrally located myonuclei were also observed in EDL (1.45% of the muscle CSA) of *Acvr1b:Tgfbr1* CKO animals and not in EDL of control animals (0.08%) (Figure 2A). Together, these data indicate that simultaneous knockout of *Acvr1b* and *Tgfbr1* results in spontaneous damage and regeneration. Spontaneous regeneration requires activation of SCs. In the low oxidative region of TA muscle of *Acvr1b:Tgfbr1* CKO animals the number of SCs per myofibre in a cross-section was increased compared to that in control animals (Figure 2B).

We next characterised cells surrounding the spontaneously regenerating regions in TA as being macrophages or fibroblasts. F4/80 staining showed that the number of macrophages per mm^2^ muscle CSA in *Acvr1b:Tgfbr1* CKO animals was increased by 14-fold (23.7 cells/mm^2^) compared to that in control (1.7 cells/mm^2^) and *Acvr1b* CKO animals (1.7 cells/mm^2^), while in *Tgfbr1* CKO animals (4.5 cells/mm^2^) the number of macrophages per mm^2^ did not differ compared to that in the other three groups (Figure 2C).

Taken together, in TA and EDL muscles that lack both *Acvr1b* and *Tgfbr1* regions with spontaneously regenerating myofibres were observed. These regions are accompanied by an increased number of SCs and macrophages.

### Lack of both *Acvr1b* and *Tgfbr1* in the skeletal myofibre increased *Hgf* expression levels and Akt/p70S6K signalling, while decreasing *Trim63* expression levels

**Figure 3.**
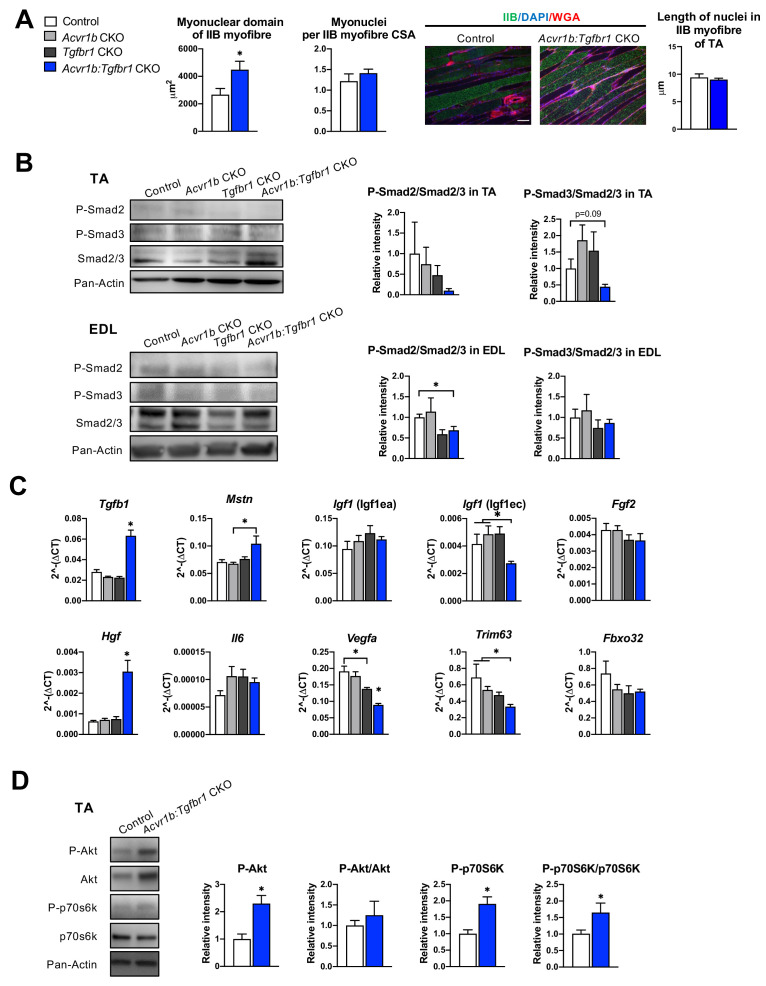
Effects of simultaneous knockout of both *Acvr1b* and *Tgfbr1* on myonuclear number and signalling for protein synthesis as well as degradation. (**A**) No differences in myonuclear lengths were observed in longitudinal sections of TA type IIB myofibres of *Acvr1b:Tgfbr1* CKO compared to control animals. This indicates that simultaneous knockout of *Acvr1b:Tgfbr1* CKO did not affect the number of myonuclei per myofibre and that the myonuclear domain (i.e. cross-sectional area/ nuclei (μm^2^)) was almost doubled. Scale bar = 100 μm. (**B**) Western blot analysis for Smad2/3 phosphorylation in TA and EDL muscle. (**C**) Relative gene expression of growth factors in non-injured muscle. (**D**) Western blot analysis of phosphorylated and total Akt and p70S6K in TA muscles. Results are presented as mean + SEM. N = 5–8 mice. *: p < 0.05. Significant differences between individual groups are indicated by lines with a *. Single * indicates significant difference compared to all other groups at the same time point. Figure 3—source data 1.Quantification of myonuclear domain, qPCR of growth factors and Western blot of P-Smad2/3, Smad2/3, P-Akt, Akt, P-p70s6k, and p70s6k in absence of injury. Figure 3—source data 2.Raw Western blot image of (**A**) P-Smad2, (**C**) P-Smad3 and (**E**) Smad2/3, (**G**) pan-Actin.Labelled images of (**B**) P-Smad2, (**D**) P-Smad3 and (**F**) Smad2/3 and (**H**) pan-Actin of tibialis anterior (TA) muscle in groups of control (con), *Acvr1b* CKO (**A**), *Tgfbr1* CKO (**T**) and *Acvr1b: Tgfbr1* CKO (dKO). Figure 3—source data 3.Raw Western blot image of (**A**) P-Smad2, (**C**) P-Smad3, and (**E**) Smad2/3, (**G**) pan-Actin.Labelled images of (**B**) P-Smad2, (**D**) P-Smad3, and (**F**) Smad2/3 and (**H**) pan-Actin of extensor digitorum longus muscle (EDL) in groups of control (con), *Acvr1b* CKO (**A**), *Tgfbr1* CKO (**T**), *Acvr1b: Tgfbr1* CKO (dKO) and positive control sample (pos). Figure 3—source data 4.Raw Western Blot image of (**A**) P-AKT, (**C**) AKT and (**E**) P-p70s6k, (**G**) p70s6k and (**I**) pan-Actin.Labelled images of (**B**) P-AKT, (**D**) AKT and (**F**) P-p70s6k, (**H**) p70s6k and (**I**) pan-Actin of TA in groups of control (con), *Acvr1b: Tgfbr1* CKO (dKO) and positive control sample (pos).

Next, we aimed to obtain insight in the mechanisms underlying the increase in *Acvr1b:Tgfbr1* CKO TA mass and myofibre CSA, as well as the observed increase in SC number and regeneration regions in these muscles. First, we determined whether the increase in myofibre size was accompanied by accretion of myonuclei. Counts of myonuclear fragments in muscle cross-sections of IIB myofibres did not differ between control and *Acvr1b:Tgfbr1* CKO animals. Moreover, in longitudinal sections no difference in length of myonuclei per myofiber was found between groups (Figure 3A), which indicates that the probability to encounter a myonucleus within a cross-section was equal between groups. The excessive hypertrophy of type IIB myofibres of *Acvr1b:Tgfbr1* CKO mice occurred without accretion of myonuclei and caused a 70% increase in the myonuclear domain (Figure 3A).

Phosphorylation of TGF-β type I receptor is known to activate canonical Smad2/3 signalling. Therefore, we examined the effects of TGF-β type I receptor knockout on Smad2/3 phosphorylation in both TA and EDL muscle (Figure 3B). Single knockout did not affect phosphorylated/total protein ratios for Smad2 and Smad3 in muscles of *Acvr1b* CKO or *Tgfbr1* CKO mice, which was in line with the lack of effect on muscle size and phenotype and suggested that at least the presence of one of the two receptors was sufficient to maintain the Smad signalling. With regard to Smad2/3 phosphorylation in TA and EDL of *Acvr1b:Tgfbr1* CKO mice, a 31% reduction was shown for phosphorylation of Smad2 in EDL while phosphorylated levels of Smads2 and 3 tended to be reduced in TA.

Since receptors were specifically knocked out in skeletal myofibres, other cell types such as SCs, fibroblasts or inflammatory cells remained sensitive to TGF-β signalling. Therefore, the effect of receptor knockout on *Tgfb1* and myostatin (*Mstn*) expression were determined. In TA muscles of *Acvr1b:Tgfbr1* CKO animals, *Tgfb1* expression levels were 2.2-fold higher compared to those of control animals, while *Tgfb1* expression levels of *Acvr1b* CKO and *Tgfbr1* CKO animals did not differ from those of control animals. *Mstn* expression levels within *Acvr1b:Tgfbr1* CKO mice were only increased compared to those of *Acvr1b* CKO animals (Figure 3C). These results indicate that lack of both *Tgfbr1* and *Acvr1b* in skeletal myofibres resulted in increased *Tgfb1* expression in muscle tissue, which may be associated with an enhanced local regeneration.

Expression levels of various growth factors, that is insulin-like growth factor 1 [*Igf1* (Igf1ea)], mechano growth factor [*Igf1* (Igf1ec)]*,* fibroblast growth factor 2 (*Fgf2*)*,* hepatocyte growth factor (*Hgf*), interleukin-6 (*Il6*) and vascular endothelial growth factor A (*Vegfa*) may contribute to SC proliferation or myofibre size (Arsic et al., 2004; Coleman et al., 1995; Lefaucheur and Sébille, 1995; Pedersen et al., 2001; Serrano et al., 2008; Tatsumi et al., 1998; Yang and Goldspink, 2002). No significant differences were observed in expression levels of *Igf1* (Igf1ea)*, Il6,* or *Fgf2. Igf1* (Igf1ec) expression levels in TA muscle of *Acvr1b:Tgfbr1* CKO animals were reduced compared to those of *Acvr1b* CKO or control animals. *Vegfa* expression levels of *Acvr1b:Tgfbr1* CKO animals were reduced compared to those of all other groups, while *Vegfa* levels in *Tgfbr1* CKO animals were reduced compared to those of control animals. In contrast, *Hgf* expression levels in *Acvr1b:Tgfbr1* CKO animals were increased compared to those in all other groups (Figure 3C). These results suggest that in TA muscle of *Acvr1b:Tgfbr1* CKO animals enhanced expression of *Hgf* may contribute to the observed increase in SC number and myofibre hypertrophy.

Myostatin and activin A both have been shown to reduce protein synthesis by decreasing phosphorylation of Akt and its downstream target p70S6 kinase (p70S6K) (Amirouche et al., 2009; Chen et al., 2014; McFarlane et al., 2006; Trendelenburg et al., 2009). In TA muscle of *Acvr1b:Tgfbr1* CKO animals, phosphorylated Akt had increased by 2.3-fold compared to that of control animals. A similar but insignificant trend was observed for total Akt relative intensity. No significant differences in the phosphorylated Akt/total Akt ratio were observed. However, phosphorylated p70S6K was increased by 1.9-fold compared to that of control animals, while total p70S6K was not significantly affected. As a consequence, the phosphorylated p70S6K/total p70S6K was increased by 1.7-fold (Figure 3D). Together, these results indicate that simultaneous knockout of *Acvr1b* and *Tgfbr1* in skeletal myofibre stimulates the protein synthesis via activation of Akt/mTOR/p70S6K signalling.

Finally, myostatin and TGF-β1 have been indicated to stimulate muscle-specific E3 ubiquitin ligases muscle RING-finger protein-1 (*Trim63*) and atrogin-1 (*Fbxo32*). *Trim63* expression levels in TA muscles of *Acvr1b:Tgfbr1* CKO mice were significantly lower compared to those of *Acvr1b* CKO or control animals. *Fbxo32* expression did not differ between groups (Figure 3C). Together, these results indicate that simultaneous knockout of *Acvr1b* and *Tgfbr1* in skeletal myofibre reduces protein breakdown via suppression of *Trim63* expression.

### Myofibre-specific receptor knockout affected the inflammatory response upon acute injury

**Figure 4.**
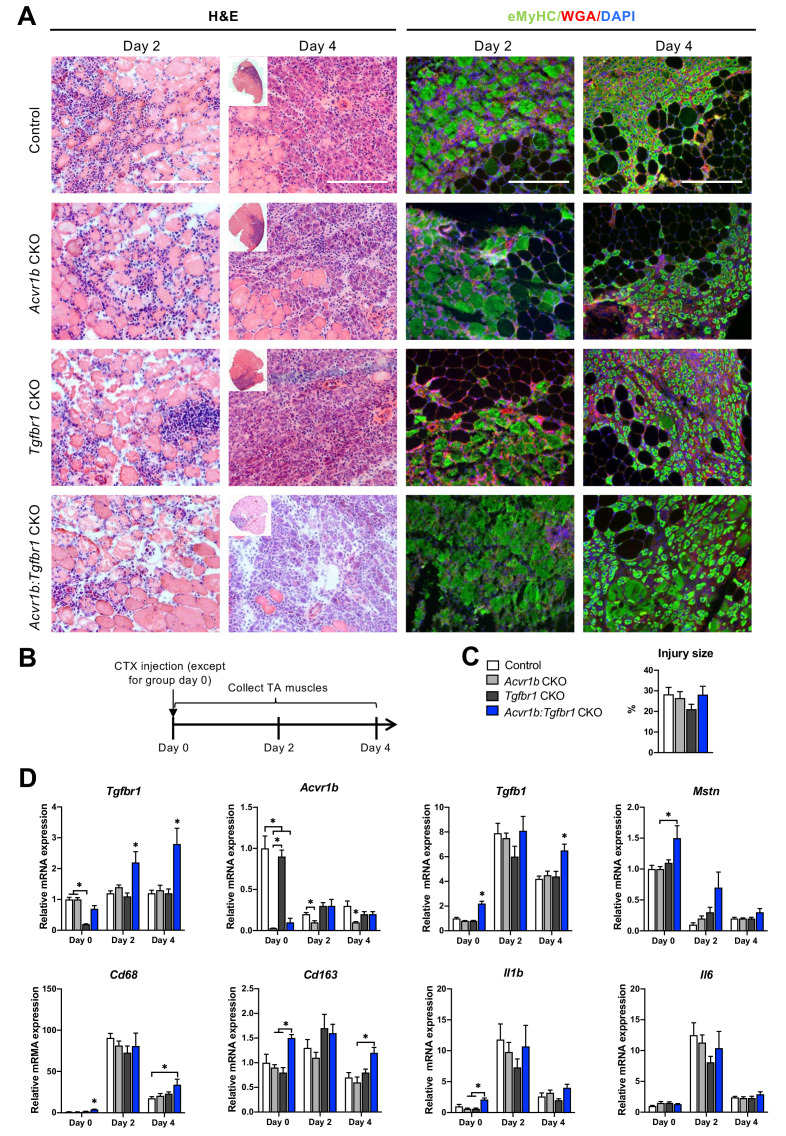
Immune response was slightly enhanced in muscle of *Acvr1b:Tgfbr1* CKO mice. (**A**) Representative images of H&E and eMyHC staining of TA sections at 2 and 4 days after CTX injection. Scale bars = 250 μm. (**B**) Scheme shows CTX injection in TA and sample collection. (**C**) Percentage of injury area was not significantly different between groups. (**D**) Relative gene expressions in TA in the absence (day 0) or presence of CTX injection after 2 and 4 days. Results are presented as mean + SEM. N = 5–8 mice, *: p < 0.05. Significant differences between individual groups are indicated by lines with a *. Single * indicates significant difference compared to all other groups at the same time point. Figure 4—source data 1.Quantification of injury size and qPCR for myogenic genes in TA at day 0,2 and 4.

After characterisation of uninjured TA muscles, effects of receptor knockout on early TA muscle regeneration were examined 2 and 4 days after CTX acute injury. Two days post injury, the injury site was characterised by increased interstitial space, indicating degradation of the endomysium, and the presence of damaged myofibres, as can be observed as unspecific green secondary antibody staining (Bencze et al., 2019). Furthermore, mononuclear cells (i.e. inflammatory cells, fibroblasts or SCs) had infiltrated the interstitial space within the injury site. Together, these observations indicate that at 2 days post injury, the inflammatory response is high and damaged myofibres have not started to regenerate yet. Four days post injury, the injury site was occupied by small, regenerating, eMyHC^+^ myofibres with centrally located nuclei. Mononuclear cells were located in the interstitial space, but the inflammatory response appeared to be reduced compared to that observed at 2 days post injury (Figure 4A). No significant differences in injury size between groups were observed (Figure 4C).

Since various cell types in muscle tissue remained sensitive to TGF-β signalling in the current model, effects of CTX injury on relative mRNA expression levels of *Tgfbr1, Acvr1b, Tgfb1,* and *Mstn* were examined. Two and 4 days post injury, relative *Tgfbr1* expression was increased in TA muscle of *Tgfbr1* CKO and *Acvr1b:Tgfbr1* CKO animals compared to day 0, which suggests that *Tgfbr1* mRNA was highly expressed in mononuclear cells (i.e. fibroblasts, inflammatory cells and SCs) that have infiltrated the injury site. For all groups, relative *Tgfb1* expression peaked at day 2 post injury. At day 4 post injury in *Acvr1b:Tgfbr1* CKO animals, *Tgfb1* expression levels remained significantly increased compared to those of other groups (Figure 4D).

At days 2 and 4, *Acvr1b* expression in *Tgfbr1* CKO and control animals decreased compared to day 0, whereas *Acvr1b* expression in *Acvr1b* CKO and *Acvr1b:Tgfbr1* CKO animals increased. Relative *Mstn* expression levels were decreased at days 2 and 4 post injury. These data suggest that under control conditions *Acvr1b* and *Mstn* are highly expressed in skeletal myofibres, while mononuclear cells that infiltrate the injury site express relatively little *Acvr1b* and *Mstn* (Figure 4D).

TGF-β1 plays an important role in the early inflammatory response after acute muscle injury. Inflammatory cells (i.e. neutrophils and macrophages), which infiltrate damaged muscle, digest cellular debris and secrete inflammatory cytokines, such as interleukin-1β (*Il1b*) and *Il6*. Here, we showed that in all groups, relative mRNA levels of macrophage-specific protein cluster of differentiation 68 (*Cd68*) (Silva et al., 1996; Smith and Koch, 1987), *Il1b* and *Il6* peaked 2 days post injury. At days 0 and 4, in TA muscle of *Acvr1b:Tgfbr1* CKO animals *Cd68* expression was increased compared to all other groups or control animals, respectively. At days 0 and 4, macrophage-specific *Cd163* (Schaer et al., 2001) expression levels of *Acvr1b:Tgfbr1* CKO animals were increased compared to those of *Acvr1b* CKO or *Tgfbr1* CKO animals (Figure 4D). At day 0 in *Acvr1b:Tgfbr1* CKO animals expression levels of *Il1b* were increased compared to those of *Acvr1b* CKO and *Tgfbr1* CKO animals. Upon injury, no differences in *Il1b* and *Il6* expression levels were observed between groups (Figure 4D). Together, these results suggest that in this model the inflammatory response peaks approximately 2 days post injury.

### Lack of both *Acvr1b* and *Tgfbr1* stimulated CSA of regenerating myofibres, myogenic gene expression and number of differentiating muscle cells during regeneration

**Figure 5.**
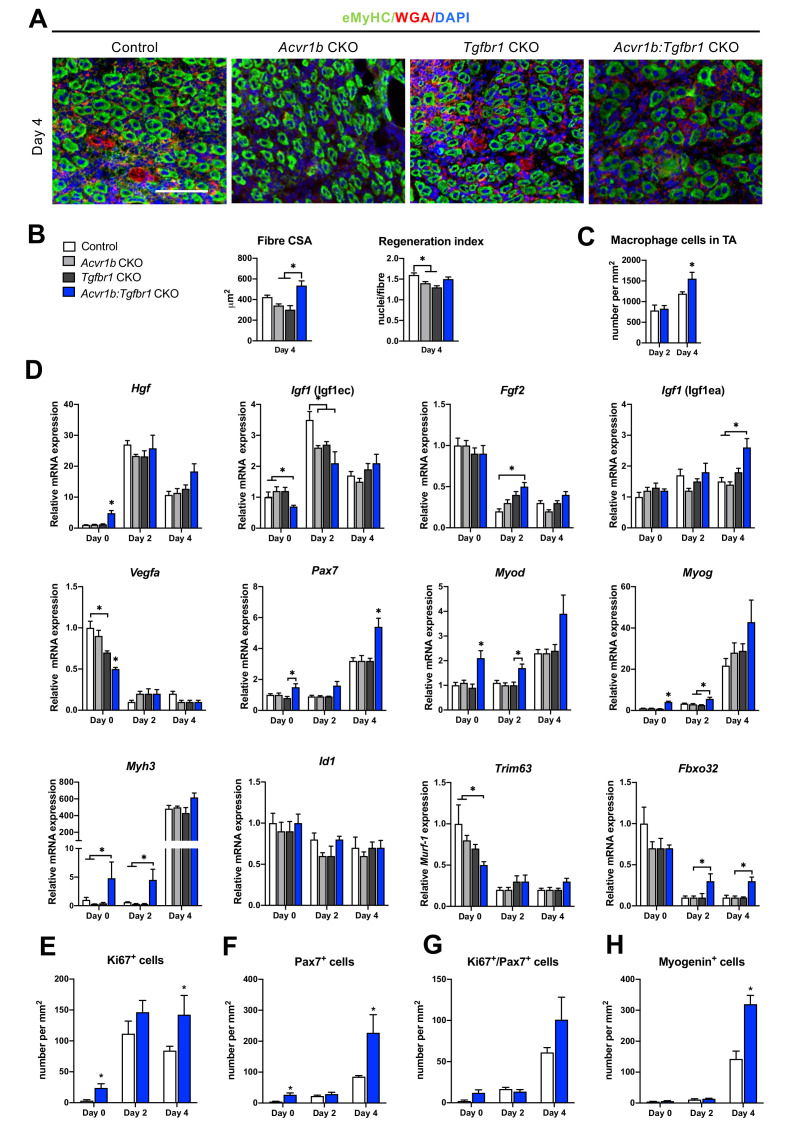
*Acvr1b:Tgfbr1* CKO mice showed enhanced CSA of regenerating myofibres and early enhanced expression of myogenic genes and differentiating cells after acute injury. (**A**) IF staining images represent eMyHC^+^ myofibres 4 days after CTX injection. Scale bar = 100 μm. (**B**) CSA of eMyHC^+^ myofibres in injured area increased in *Acvr1b:Tgfbr1* CKO mice compared to *Acvr1b* CKO and *Tgfbr1* CKO animals, while RI was decreased in both *Acvr1b* CKO and *Tgfbr1* CKO mice compared to controls. (**C**) Number of macrophages was quantified in the injured area. (**D**) Relative gene expression in TA in absence (day 0) or presence of CTX injection after 2 and 4 days are presented. Increased number of Ki67^+^ cells (**E**) and Pax7^+^ (**F**) cells were found in TA of *Acvr1b:Tgfbr1* CKO mice in absence of injury as well as 4 days after CTX injection. (**G**) Four days post injury, number of Ki67^+^/Pax7^+^ cells was not different between control and *Acvr1b:Tgfbr1* CKO mice. (**H**) More Myogenin^+^ cells were found in injured area of *Acvr1b:Tgfbr1* CKO mice on day 4 post injury. Results are presented as mean + SEM. N = 5–8 mice, *: p < 0.05. Significant differences between individual groups are indicated by lines with a *. Single * indicates significant difference compared to all other groups at the same time point. Figure 5—source data 1.Quantification of regenerating myoblasts upon acute injury, qPCR results and number of myogenic committed cells in TA at day 0,2 and 4.

Effects of receptor knockout on muscle regeneration after acute injury were examined (Figure 5A). In *Acvr1b:Tgfbr1* CKO animals CSA of regenerating myofibres was increased compared to *Acvr1b* CKO and *Tgfbr1* CKO animals, but not compared to controls (Figure 5B). Regeneration index (RI) was reduced in muscle tissue of *Acvr1b* CKO and *Tgfbr1* CKO animals compared to that of control animals, while RI of *Acvr1b:Tgfbr1* CKO animals was not significantly different compared to that of other three groups (Figure 5B). Next, we hypothesized an increased immune response was involved in the accelerated muscle regeneration process after cardiotoxin induced muscle injury in the absence of *Acvr1b* and *Tgfbr1*. Macrophages were identified by F4/80 in IF staining (Figure 5—figure supplement 1). The number of macrophages in TA of *Acvr1b:Tgfbr1* CKO animals at day 4 post injury was significantly increased compared to that in control animals (Figure 5C). Taken together, after acute injury individual knockout of *Acvr1b* or *Tgfbr1* expression in mature myofibre reduced myofibre regeneration, while simultaneous knockout of *Acvr1b* and *Tgfbr1* stimulated this which was accompanied by increased infiltration of macrophages.

**Figure 5—figure supplement 1.**
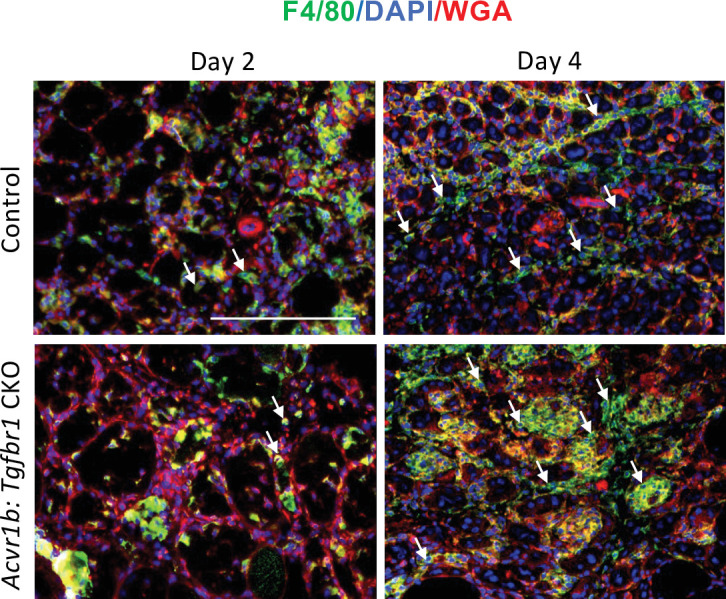
Number of macrophages in TA of *Acvr1b:Tgfbr1* CKO animals was increased 4 days post injury. Number of macrophages (F4/80^+^, green) in control and *Acvr1b:Tgfbr1* CKO animals on days 2 and 4 post injury. Nuclei were stained by DAPI (blue) and ECM of muscle were stained by WGA (red). Scale bar = 100 μm. N = 5–7.

**Figure 5—figure supplement 2.**
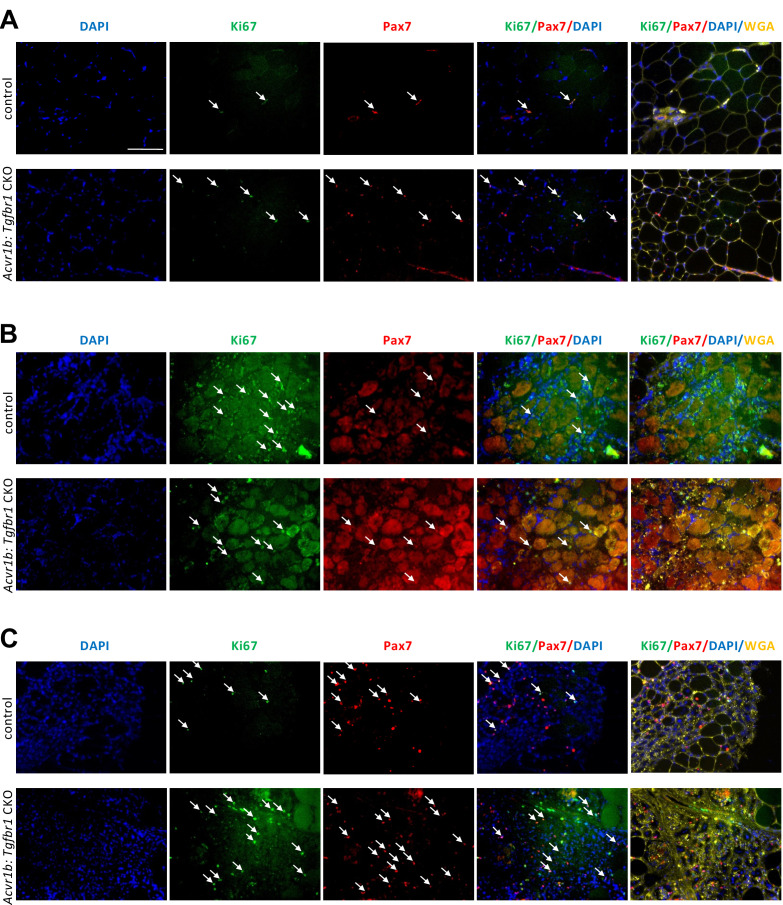
Increased number of proliferating cells and satellite cells in TA of *Acvr1b:Tgfbr1* CKO animals in absence of injury and 4 days post injury. (**A**) In absence of injury, proliferating cells (Ki67^+^, green, white arrows) and muscle satellite cells (Pax7^+^, red, white arrows) were shown by IF staining in low oxidative area of TA, where regeneration patches were found. Two (**B**) and 4 (**C**) days post injury, more Ki67^+^ and Pax7^+^ cells (white arrows) infiltrated in injured area. Nuclei were stained by DAPI (blue) and ECM of muscle were stained by WGA (yellow). Scale bar = 100 μm. N = 5–7.

**Figure 5—figure supplement 3.**
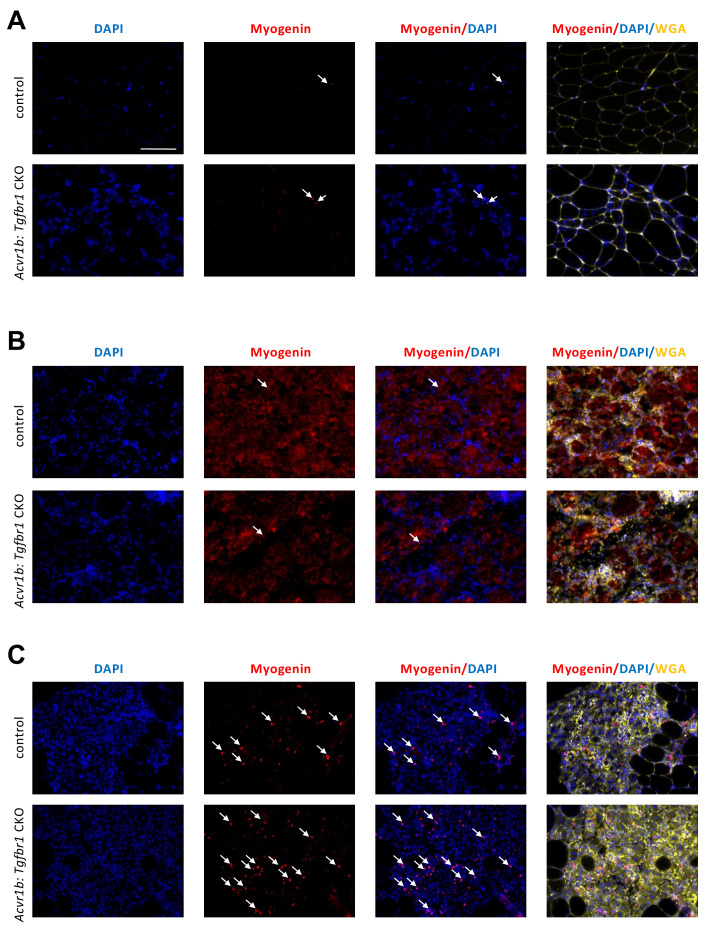
Increased number of differentiating muscle cells in TA of *Acvr1b:Tgfbr1* CKO animals 4 days post injury. On day 0 (**A**) and 2 days (**B**) post injury, the number of differentiating myoblasts (myogenin^+^, red, white arrows) was of no difference between groups. Four days (**C**) post injury, more Myogenin^+^ cells were found in injured area of *Acvr1b:Tgfbr1* CKO animals. Nuclei were stained by DAPI (blue) and ECM of muscle were stained by WGA (yellow). Scale bar = 100 μm. N = 5–7.

The effects of receptor knockout on expression of genes involved in SC activation, differentiation and muscle growth were analysed in order to understand observed differences in RI and CSA of regenerating myofibres. First of all, mRNA expression levels of various growth factors were differently affected by receptor knockout. *Hgf* and *Igf1* (Igf1ec) expression peaked at day 2 post injury. At day 2 in *Acvr1b* CKO and *Acvr1b:Tgfbr1* CKO mice *Igf1* (Igf1ec) expression was lower compared to control animals. At day 4 in *Acvr1b:Tgfbr1* CKO animals *Igf1* (Igf1ea) levels were increased compared to those of *Acvr1b* CKO and control animals. *Vegfa* and *Fgf2* expression levels were decreased after injury. At day 2 in *Acvr1b:Tgfbr1* CKO mice *Fgf2* expression was increased (Figure 5D). Together, these results indicate that in simultaneous receptor knockout enhanced *Igf1* (Igf1ea) and *Fgf2* expression post injury contribute to the accelerated early regeneration.

Proper muscle regeneration is regulated by sequential expression of myogenic genes. Thus, relative mRNA expression levels of myogenic genes (i.e. paired box protein 7 (*Pax7*), myoblast determination protein 1 (*Myod*), myogenin (*Myog*), muscle embryonic myosin heavy chain (*Myh3*) as well as inhibitor of differentiation 1 (*Id1*)), were examined. At day 4, in all groups expression of *Pax7, Myod, Myog* and *Myh3* had increased. At day 0 in *Acvr1b:Tgfbr1* CKO mice, *Pax7* expression was increased compared to that of *Tgfbr1* CKO mice, while at day 4 in *Acvr1b:Tgfbr1* CKO mice *Pax7* expression was increased compared to that in all other groups. At day 0 in *Acvr1b:Tgfbr1* CKO mice *Myod* and *Myog* expression levels were increased compared to those in other groups, while at day 2 in *Acvr1b:Tgfbr1* CKO mice *Myod* expression levels were increased compared to those in *Tgfbr1* CKO mice and *Myog* expression levels were increased compared to those in both *Acvr1b* CKO and *Tgfbr1* CKO animals. At day 4 post injury, no differences in *Myod* or *Myog* expression levels were observed between groups, although a trend suggested that expression of both genes was increased in *Acvr1b:Tgfbr1* CKO mice. At both day 0 and day 2, in *Acvr1b:Tgfbr1* CKO mice *Myh3* levels were increased compared to those in both *Acvr1b* CKO and control animals. At day 4 post injury, no differences in *Myh3* mRNA expression levels were observed between groups (Figure 5D). Receptor knockout did not affect *Id1* expression. Taken together, these results show that in TA myofibre-specific *Acvr1b* and *Tgfbr1* receptor knockout stimulates myogenic gene expression.

Lastly, relative expression levels of muscle-specific E3 ligases Trim63 (*Trim63*) and atrogin-1 (*Fbxo32*) during early regeneration were considered. *Trim63* and *Fbxo32* are expressed in mature myofibres, but not in SCs and inhibit myofibre growth and hypertrophy. For *Acvr1b* CKO, *Tgfbr1* CKO and control animals, at day 4 *Trim63* levels were decreased compared to those at day 0, while for *Acvr1b:Tgfbr1* CKO animals no significant differences in *Trim63* expression were observed over time. At both day 2 and 4 post injury, in *Acvr1b:Tgfbr1* CKO animals *Fbxo32* expression levels were relatively increased compared to those in *Acvr1b* CKO mice, while no differences in *Trim63* expression levels were observed between groups (Figure 5D). Together, these results indicate E3 ligases do not play a role in the observed increase in size of regenerating myofibres.

On day 0, the number of proliferating cells (Ki67^+^) in low oxidative region of TA of *Acvr1b:Tgfbr1* CKO animals was about 7.6-fold higher than that in control animals (Figure 5E). Two days after injury, a 6-fold increase of proliferating cells was found in *Acvr1b:Tgfbr1* CKO animals compared to that on day 0. Moreover, at day 4 after injury, the number of proliferating cells in *Acvr1b:Tgfbr1* CKO animals was 1.7-fold higher than that in control animals. To determine whether the increased CSA of regenerating myofibres in *Acvr1b:Tgfbr1* CKO animals was due to an increased SCs number and advanced differentiation of myoblasts, we tested SCs proliferation and activation status. Although at day 0 and 4, the number of SCs (Pax7^+^) cells was higher in *Acvr1b:Tgfbr1* CKO animals than in control animals (Figure 5F), the number of proliferating SCs (Ki67^+^/Pax7^+^) did not differ from that in control (Figure 5G, Figure 5—figure supplement 2). Nevertheless, an accelerated rate of increase in Ki67^+^/Pax7^+^ cells was shown. Note that, at day 4 after injury the number of myogenin^+^ cells was more than 2.2-fold higher in *Acvr1b:Tgfbr1* CKO animals (Figure 5H, Figure 5—figure supplement 3). These findings indicate that muscle regeneration upon acute injury was improved in *Acvr1b:Tgfbr1* CKO animals, which was attributed to an accelerated myogenic process.

### Simultaneous knockout of both *Acvr1b* and *Tgfbr1* within the myofibre enhanced ECM deposition

**Figure 6.**
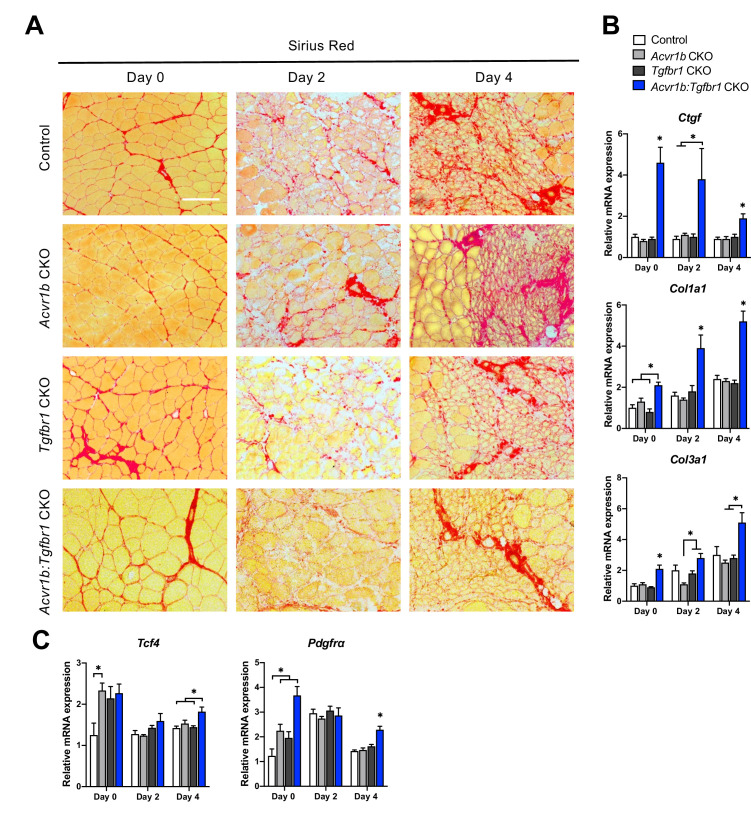
Relative mRNA expression levels of ECM components were enhanced in *Acvr1b:Tgfbr1* CKO mice. (**A**) Sirius Red staining shows collagen deposition in absence (day 0) or presence of CTX injection after 2, and 4 days (scale bar = 100 μm). (**B, C**) Relative gene expression in TA muscle in absence (day 0) or presence of CTX injection after 2 and 4 days. Results are presented as mean + SEM. N = 5–8 mice, *: p < 0.05. Significant differences between individual groups are indicated by lines with a *. Single * indicates significant difference compared to all other groups at the same time point. Figure 6—source data 1.Quantification of qPCR for extracellular matrix genes in TA at day 0,2 and 4.

Another essential aspect of muscle regeneration is connective tissue remodelling. Figure 5A shows Sirius Red stainings at different stages of regeneration. At day 0, myofibres were surrounded by a thin layer of endomysium. At 2 days post injury, this endomysium appeared to be disrupted for a large part. At day 4 post injury, a large amount of connective tissue was observed surrounding the regenerating myofibres (Figure 6A).

Effects of myofibre-specific *Acvr1b* and *Tgfbr1* receptor knockout on ECM remodelling were assessed by examining connective tissue growth factor (*Ccn2*), collagen type 1, alpha 1 (*Col1a1*) and collagen type 3, alpha 1 (*Col3a1*) expression. At all time points, in *Acvr1b:Tgfbr1* CKO animals *Ccn2* and *Col1a1* mRNA expression levels were substantially increased compared to those of control animals or all groups. At day 0, in *Acvr1b:Tgfbr1* CKO animals, *Col3a1* expression levels were increased compared to those in other groups. At days 2 and 4, in *Acvr1b:Tgfbr1* CKO animals *Col3a1* expression was increased compared to *Acvr1b* CKO or both *Acvr1b* CKO and *Tgfbr1* CKO animals (Figure 6B).

To determine whether the number of fibroblasts was increased in TA muscles by knockout of *Acvr1b* and *Tgfbr1*, relative mRNA expression levels of fibroblast markers, transcription factor 4 (*Tcf4*) and platelet-derived growth factor receptor A (*Pdgfra*) (Mathew et al., 2011) were determined. At day 0, *Tcf4* expression levels of TA in *Acvr1b* CKO mice were increased compared to those in control animals, but were not different from those in *Tgfbr1* CKO or *Acvr1b:Tgfbr1* CKO animals. In *Acvr1b:Tgfbr1* CKO animals, relative *Pdgfra* expression levels were increased compared to those of control and *Acvr1b* CKO animals. Noteworthy, 4 days post injury, both *Tcf4* and *Pdgfra* mRNA levels were increased in *Acvr1b:Tgfbr1* CKO animal compared to those in control mice (Figure 6C).

## Discussion

**Figure 7.**
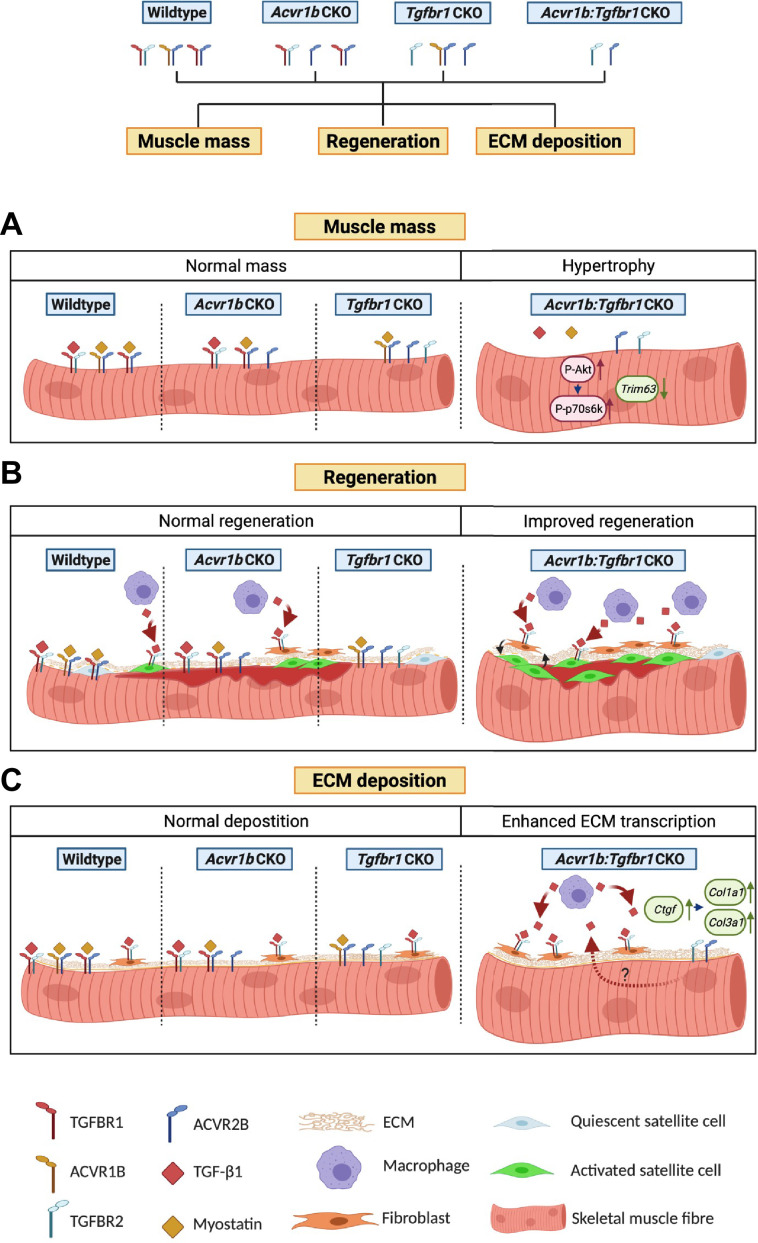
Schematic diagram of the effects of single or combined muscle-specific knockout of *Tgfbr1* and/or *Acvr1b* receptors on muscle hypertrophy, regeneration, and expression of ECM components. (**A**) Myofibre size is not affected after individual knockout of *Acvr1b* or *Tgfbr1*, which indicates that these receptors have redundant effects on muscle size and that myostatin signals via both receptors to control muscle mass. Simultaneous knockout of both *Acvr1b* and *Tgfbr1* inhibits signaling of TGF-β, myostatin and activin A and stimulates protein synthesis via the Akt/mTOR/p70S6K pathway, while inhibiting protein breakdown through repression of *Trim63* levels, resulting in substantial muscle hypertrophy. (**B**) Upon acute injury, simultaneous knockout of combined *Acvr1b* and *Tgfbr1* accelerates early muscle regeneration, as observed by increased myogenic gene expression as well as increased CSA of regenerating myofibres. An increased number of SCs likely contributes to these effects. (**C**) Simultaneous myofibre-specific knockout of *Acvr1b* and *Tgfbr1* induces mRNA expression of ECM components. These effects are likely caused by enhanced TGF- β1 signaling in fibroblasts. Schematic is created using BioRender.

The aim of this study was to investigate effects of mature myofibre-specific knockout of type I receptors *Tgfbr1* and *Acvr1b* on muscle morphology as well as early muscle regeneration, inflammation and collagen deposition in both uninjured muscle tissue and after acute CTX injury. We observed that simultaneous knockout of *Acvr1b* and *Tgfbr1* resulted in a substantial increase in TA and EDL muscle mass as well as type IIB myofibre CSA. *Tgfbr1* knockout only marginally increased muscle mass, while *Acvr1b* knockout did not affect muscle mass. In the low oxidative region of TA muscle tissue of *Acvr1b:Tgfbr1* CKO animals the percentage of type IIB myofibres was reduced, while in EDL no differences in myofibre type distribution were observed. Remarkably, simultaneous knockout of both *Acvr1b* and *Tgfbr1* caused spontaneous regeneration and an increase in SC number in the low oxidative region of TA and in EDL muscle tissue. Lack of both *Acvr1b* and *Tgfbr1* in the skeletal myofibre of TA increased myofibre CSA of regenerating myofibres, number of regenerating cells and macrophages during regeneration, as well as enhanced expression levels of myogenic gene and growth factors. In both uninjured and regenerating muscles, simultaneous knockout of *Acvr1b* and *Tgfbr1* in the myofibre resulted in increased ECM and fibroblast gene expression. Figure 7 shows a schematic summarising the main results.

Together, these results indicate that simultaneous receptor knockout stimulates muscle hypertrophy and promotes early muscle regeneration upon injury, whereas individual receptor knockout does not.

### Simultaneous knockout of *Acvr1b* and *Tgfbr1* induces hypertrophy by both inhibiting protein degradation and stimulating protein synthesis

Our data show that simultaneous myofibre-specific knockout of both *Acvr1b* and *Tgfbr1* is required for muscle hypertrophy, while inhibition of *Acvr1b* does not affect muscle mass or myofibre size and inhibition of *Tgfbr1* has only marginal effects. Supporting our data, a recent study showed that simultaneous inhibition of *Acvr1b* and *Tgfbr1* was required to enhance muscle mass, while individual receptor inhibition had little effect (Lee et al., 2020). These results indicate that *Acvr1b* and *Tgfbr1* have redundant functions in the regulation of muscle mass.

Myostatin and activin A negatively regulate muscle mass by stimulating protein degradation through upregulation of E3 ligases and reducing protein synthesis through a decrease in phosphorylation of Akt and its downstream target p70S6K (Amirouche et al., 2009; Chen et al., 2014; McFarlane et al., 2006; Trendelenburg et al., 2009). TGF-β overexpression in vivo has been suggested to increase *Fbxo32* expression and concomitantly cause muscle atrophy (Mendias et al., 2012). Simultaneous receptor knockout increased relative expression of phosphorylated Akt and lack of both *Acvr1b* and *Tgfbr1* increased the phosphorylated p70S6K/total p70S6K ratio, which indicates increased protein synthesis via the Akt/mTOR/p70S6K pathway contributes to muscle hypertrophy.

Simultaneous knockout of *Acvr1b* and *Tgfbr1* also reduced mRNA expression levels of E3 ligases *Trim63*, indicating reduced protein degradation. Simultaneous knockout of *Acvr1b* and *Tgfbr1* increased *Hgf* expression levels. HGF signaling has been shown to protect skeletal muscle against atrophy after denervation or in muscle pathology by reducing relative *Trim63* and *Fbxo32* expression levels (Choi et al., 2018). Together, these results indicate that simultaneous knockout of both *Acvr1b* and *Tgfbr1* both decrease protein breakdown and stimulate protein synthesis.

The TGF-β type I receptor knockout induced an increase in Akt signalling and reduction in E3 ligase expression are likely mediated at least in part via elevated *Hgf* expression levels in myofibres. In both myoblasts and myotubes, HGF stimulates the Akt/mTOR pathway (Chen et al., 2012; Perdomo et al., 2008). In addition, reduced Smad2/3 phosphorylation within myofibres likely contributes to the increase in Akt signalling and reduction in E3 ligase expression (Goodman et al., 2013; Sartori et al., 2009). In TA and EDL of *Acvr1b:Tgfbr1* CKO animals, Smad2/3 phosphorylation was or tended to be reduced, respectively. Note that TGF-β type I receptors were specifically knocked out in myofibres and that Smad2/3 in various other cell types could still be phosphorylated by TGF-β1, myostatin and activin A, masking the changes in myofibres.

TGF-β1, myostatin and activin A regulate skeletal muscle mass via similar mechanisms. Previous research has shown that inhibition of myostatin and to a lesser extent activin A is sufficient to induce muscle hypertrophy (Wu et al., 2017). Here, we show that inhibition of TGF-β1 or activin A signaling via their type I receptor is insufficient to induce muscle hypertrophy. In muscle myostatin likely signals via both type I receptors to regulate muscle mass. In muscles that lack either *Acvr1b* or *Tgfbr1,* we observed no changes in Smad2/3 signaling, which was in accordance with the observation there was no or modest effect on muscle hypertrophy. Targeting both receptors is indispensable to substantially reduce TGF-β1/myostatin/activin A signalling and induce muscle hypertrophy.

### Simultaneous knockout of *Acvr1b* and *Tgfbr1* specifically enhances type IIB myofibre CSA without accretion of myoblasts

Simultaneous knockout of *Acvr1b* and *Tgfbr1* most substantially increased type IIB myofibre CSA. This is likely the result of myofibre type-related hypertrophic capacity rather than myofibre type-specific receptor knockout bias (McCarthy et al., 2012b). It has been suggested that mainly fast twitch myofibres possess the ability to hypertrophy, while slow twitch myofibres are unlikely to increase in size (van Wessel et al., 2010). Additionally, ACVR2B is more abundantly expressed in type II than type I myofibres, thus a more substantial effect on myofibre hypertrophy was expected upon type I receptor depletion (Babcock et al., 2015). Remarkably, type IIB myofibre hypertrophy occurred without apparent accretion of myonuclei, which resulted in an approximately 70% increase in myonuclear domain. The lack of difference in the number of myonuclei per myofibre cross-section together with the lack of difference in myonuclear length in type IIB myofibres of TA indicates that the total number of nuclei per myofibre was not affected by simultaneous knockout of *Acvr1b* and *Tgfbr1*. Although for example exercise induced hypertrophy is often accompanied by increased myonuclei number (Conceição et al., 2018; van der Meer et al., 2011), the myonuclear domain is known to be flexible and increases in myonuclear domain of 30% have been reported (Murach et al., 2018). Moreover, inhibition of myostatin signalling using soluble ACVR2B leads to hypertrophy without accretion of SCs (Lee et al., 2012). Here, we show that in type IIB myofibres myonuclear domain can increase by at least 70%, without requirement of accretion of myonuclei to sustain myofibre growth. To the best of our knowledge such increase in myonuclear domain has not been reported before.

Since myonuclei are required for mitochondrial biogenesis, a local reduction in oxidative capacity was expected (Hock and Kralli, 2009; Kotiadis et al., 2014). In this study, knockout of both *Acvr1b* and *Tgfbr1* resulted in decreased SDH activity in the low oxidative region of TA. Previous research has shown that an inverse relation exists between myofibre CSA and oxidative capacity (van der Laarse et al., 1989), whereas myofibre CSA is positively correlated to glycolytic capacity (Rivero et al., 1998). Recent evidence suggests that, similar to ‘Warburg effect’ in tumours, in hypertrophying skeletal muscle reprogramming towards a more glycolytic metabolism occurs. Glycolytic enzyme pyruvate kinase muscle isoform 2 (PKM2), which is particularly highly expressed in type II myofibres, contributes to the increased hypertrophic potential of type II myofibres (Verbrugge et al., 2020).

Although lack of *Acvr1b* and *Tgfbr1* decreased SDH activity, integrated SDH activity (SDH activity times CSA) was increased. Previous research has shown that integrated SDH activity correlates with the maximal rate of oxygen consumption (VO_2max_) and mitochondrial density, which suggests that the total oxidative capacity of these myofibres in *Acvr1b:Tgfbr1* CKO animals was increased (van der Laarse et al., 1989). Present data show that by targeting both receptors simultaneously it is possible to deviate from the tight relation between myofibre size and oxidative metabolism (i.e. simultaneous increases in both myofibre size and oxidative capacity). The role of both receptors in the synthesis of mitochondria warrants further investigation.

### Lack of both *Acvr1b* and *Tgfbr1* reduces the percentage of type IIB myofibres within the low oxidative region of the TA

Another remarkable finding was the reduction in the percentage of type IIB myofibres in the low oxidative region of the TA muscle of *Acvr1b:Tgfbr1* CKO animals. In the high oxidative region of the TA muscle as well as the EDL, no differences in myofibre type distribution were observed. In contrast to our findings, previous research has shown increased percentage of fast, glycolytic myofibres in skeletal muscle of *Mstn^-/-^* mice (Amthor et al., 2007; Girgenrath et al., 2005; Hennebry et al., 2009). Moreover, increased myostatin/activin A signalling in follistatin mutant mice showed increased the percentage of slow, oxidative myofibres (Lee et al., 2010). Note that in a genetic knockout mouse model, absence of myostatin precedes myogenesis and may influence skeletal muscle development, whereas in our model TGF-β signalling was inhibited in mature skeletal muscle. The reduction in type IIB myofibres may also be a consequence of local damage to the myofibres, rather than a phenotypical change caused by receptor knockout. Taken together, these results indicate that type I receptor knockout in mature myofibres has minor effects on myofibre type distribution.

### Simultaneous knockout of *Acvr1b* and *Tgfbr1* in myofibre may result in accelerated early regeneration

We observed that simultaneous knockout of *Acvr1b* or *Tgfbr1* increased the CSA of regenerating myofibres compared to individual receptor knockout, while a trend was visible compared to controls. In addition, at day 0, simultaneous receptor knockout enhanced *Hgf* expression as well as the number of SCs per myofibre and concomitantly relative expression levels of *Pax7* and *Myod,* indicating that SCs were activated prior to CTX injection. Muscle regeneration is dependent on activation of Pax7^+^ SCs and sequential expression of myogenic genes (Charge and Rudnicki, 2004; Delaney et al., 2017; Ishido and Kasuga, 2011; Lepper et al., 2011). HGF is the primary growth factor for SC activation and may have accelerated early muscle regeneration (Allen et al., 1995; Gal-Levi et al., 1998; Miller et al., 2000; Tatsumi et al., 1998). Receptor knockout did not affect *Hgf* expression after injury, which indicates HGF expression and subsequent SC activation is likely not induced by lack of TGF-β signalling in the myofibre, but rather a consequence of spontaneous damage and regeneration.

Moreover, 2 or 4 days post injury simultaneous knockout of *Acvr1b* and *Tgfbr1* increased expression levels of *Fgf2* or *Igf1* (Igf1ea). Simultaneous overexpression of IGF-1 and FGF-2 has a synergistic effect on both myoblast proliferation as well as fusion index (Allen and Boxhorn, 1989). Thus, simultaneous knockout of *Acvr1b* and *Tgfbr1* in skeletal myofibre likely enhances early muscle regeneration via enhanced expression of *Igf1* (Igf1ea) and *Fgf2*. However, myogenic gene expression was increased prior to *Igf1* (Igf1ea) and *Fgf2* upregulation, which indicates that although these growth factors may positively contribute to early regeneration, upregulation of *Igf1* (Igf1ea) and *Fgf2* cannot fully explain effects on early regeneration.

Simultaneous receptor knockout did not reduce mRNA expression of E3 ligases during regeneration, indicating that the increase in myofibre CSA is not caused by reduced protein breakdown. In contrast, during regeneration *Fbxo32* expression is enhanced in *Acvr1b:Tgfbr1* CKO animals compared to other groups, but here the enhanced *Fbxo32* levels may correspond with muscle regeneration.

Moreover, for *Acvr1b:Tgfbr1* CKO animals the numbers of SCs and differentiating myoblasts within the injured regions 4 days post injury were more than doubled compared to those in control animals. The observation that at all time points the number of proliferating SCs (i.e. Ki67^+^/Pax7^+^ cells) was not different between control and *Acvr1b:Tgfbr1* CKO animals indicates that in the *Acvr1b:Tgfbr1* CKO animals muscle damage had initiated activation and proliferation in the injured region between days 2 and 4. Alternatively, SCs had migrated from adjacent myofibres to the site of injury or from intact regions along the myofibres (Ishido and Kasuga, 2011; Schultz et al., 1985). The accelerated myoblast proliferation and differentiation likely contributed to the enhanced protein synthesis and hypertrophy of newly formed myofibres.

A limitation of this study is that we did not observe later stages of muscle regeneration. Additional research is required to determine whether changes observed in this study ultimately result in a shorter regeneration period.

### *Acvr1b* and *Tgfbr1* affect inflammatory response after injury

Proper activation of the immune response and expression of inflammatory cytokines is important for myoblast proliferation and myogenic gene expression during early muscle regeneration (Cantini et al., 1995; Chaweewannakorn et al., 2018; Grabiec et al., 2013; Zhang et al., 2013). In absence of injury, compared with control, 20-fold higher number of macrophages was found the in low oxidative area of TA in *Acvr1b:Tgfbr1* CKO animals, indicating increased immune cell residence at baseline before CTX injury. Two days post injury a large infiltration of mononucleated cells was observed in all groups, as well as a peak in relative expression levels of *Tgfb1*, *Cd68*, *Il1b,* and *Il6*.

Macrophages play an important role in the regulation of muscle regeneration (Tidball, 2017). Macrophages are classified in M1 (pro-inflammatory) and M2 (anti-inflammatory) macrophages (Mosser and Edwards, 2008). Early after injury, gene expression of pan-macrophages marker *Cd68* and M2 macrophage marker *Cd163* (Hu et al., 2017), as well as the number of macrophages was increased in both control and *Acvr1b:Tgfbr1* CKO animals. Expression levels of *Il6* and *Il1b*, which are typical cytokines expressed by M1 macrophages, were not higher than in control animals, while *Igf1 (Igf1ea*) expression, also known to be expressed by M1 macrophages, was increased in *Acvr1b:Tgfbr1* CKO animals which was likely advantageous to expand the SC pool and to induce hypertrophy of newly formed myofibres. Moreover, TGF-β1 expression was increased in muscle with simultaneous knockout of *Acvr1b:Tgfbr1* CKO. At a later stage after injury, M2 macrophages are known to promote myogenic differentiation and stimulate ECM deposition by releasing TGF-β1 (Arnold et al., 2007; Novak et al., 2014). Taken together, we conclude that muscle-specific lack of both receptors promotes an inflammatory response by enhanced infiltration of macrophages which is associated with accelerated muscle regeneration.

### Lack of both *Acvr1b* and *Tgfbr1* enhances gene expression of ECM components in both intact and injured TA muscle

In both uninjured TA muscle tissue, as well as after CTX injury simultaneous knockout of *Tgfbr1* and *Acvr1b* in skeletal myofibre increased *Ccn2*, *Col1a1* and *Col3a1* mRNA expression. These increases in gene expression were conceivably caused by TGF-β signalling in other cell types present within the muscle tissue, that is fibroblasts. This hypothesis is supported by the infiltration of fibroblasts in TA muscle of *Acvr1b:Tgfbr1* CKO animals in the absence of injury, as well as increased gene expression levels of *Pdgfra*. Furthermore, after injury in *Acvr1b:Tgfbr1* CKO animals *Tgfbr1, Tcf4* and *Pdgfra* levels were increased compared to those in other groups, which indicated increased infiltration of non-muscle cells upon injury. Together these results support the hypothesis that TGF-β and myostatin act on fibroblasts and possibly other cell types within the muscle tissue to induce expression of collagens. We previously showed that inhibition of *Tgfbr1* in C2C12 myoblasts reduced *Ccn2* and *Col1a1* expression (Hillege et al., 2020). Moreover, systemic administration of anti-TGF-β or soluble ACVR2B in murine X-linked muscular dystrophy (mdx) mice reduced muscular fibrosis (Andreetta et al., 2006; Bo Li et al., 2012). In conclusion, to reduce expression of ECM components within skeletal muscle inhibition of TGF-β signalling in other cell types such as fibroblasts and satellite cells is required.

Chronic excessive ECM deposition leads to increased muscle stiffness and loss of function. However, transiently enhanced ECM deposition is essential to early muscle regeneration and results in scar free muscle repair in various types of acute injury (Hardy et al., 2016; Mahdy et al., 2015). In this study after CTX injury *Col1a1* and *Col3a1* expression increased in all groups. Transient enhanced ECM deposition is required to maintain muscle structural integrity and provides a scaffold for regenerating myofibres (Kääriäinen et al., 2000). Furthermore, interaction between fibroblasts and SCs appears to be essential for proper muscle regeneration, since fibroblasts prevent early differentiation of SCs, while in turn SCs control the number of fibroblasts (Murphy et al., 2011b). Thus the observed enhanced expression of ECM components in TA that lacks both receptors may contribute to the increased number of SCs at day 0 and acceleration of early muscle regeneration.

### Implications for ACVR1B and TGFBR1 inhibition as potential therapeutic strategy

An important limitation of our study is that we investigated effects of *Acvr1b* and *Tgfbr1* knockout on early regeneration after an acute injury. In contrast to our model, a dystrophic or aged mouse model has characteristics such as chronic inflammation, impaired regeneration and fibrosis. Further research is required to determine how *Acvr1b* and *Tgfbr1* knockout affects long-term regeneration capacity, chronic inflammation, and fibrosis in a pathological model.

Our data indicate that simultaneous knockout of *Acvr1b* and *Tgfbr1* in the mature myofibre causes muscle hypertrophy and accelerates early muscle regeneration. The inflammatory response is likely independent on TGF-β signalling within the myofibre. Furthermore, specifically targeting *Tgfbr1* and *Acvr1b* in mature myofibre actually increased relative ECM gene expression levels. This effect is likely the result of enhanced TGF-β signalling in other cell types (i.e. fibroblasts and inflammatory cells). Thus, targeting TGF-β signalling in immune cells and fibroblasts present in muscle tissue is likely required to alleviate chronic inflammation and fibrosis.

Taken together, our data indicate that individually inhibiting either *Acvr1b* or *Tgfbr1* may not be sufficient to alleviate muscle pathologies, nevertheless combined inhibition of both *Acvr1b* and *Tgfbr1* may increase muscle mass and accelerate early muscle regeneration.

## Materials and methods

**Key resources table:** 

Reagent type (species) or resource	Designation	Source or reference	Identifiers	Additional information
Strain, strain background (C57BL/6, males)	C57BL/6	PMID:22564549	# 025750	Jackson Laboratory, Bar Harbor, ME, USA
Genetic reagent (Transfected construct (*Mus musculus*))	HSA-Cre	PMID:22564549	# 025750	Jackson Laboratory, Bar Harbor, ME, USA
Genetic reagent (Transfected construct (*Mus musculus*))	Acvr1b^fl/fl^	PMID:23109354		Cancer Research Center of Lyon, French Institute of Health and Medical Research
Genetic reagent (Transfected construct (*Mus musculus*))	Tgfbr1^fl/fl^	PMID:11285230		Leiden University Medical Center
Antibody	Anti-phospho-Smad2 (Ser465/467) (138D4) (Rabbit monoclonal)	Cell Signaling Technology	Cat# 3108, RRID:AB_490941	WB (1:500)
Antibody	Anti-phospho-Smad3 (Ser423/425) (C25A9) (Rabbit monoclonal)	Cell Signaling Technology	Cat# 9520, RRID:AB_10203253	WB (1:500)
Antibody	Anti-Smad2/3 (Mouse monoclonal)	Cell Signaling Technology	Cat# 610843, RRID: AB_398162	WB (1:500)
Antibody	Anti-phospho-AKT (Ser473) (Rabbit monoclonal)	Cell Signaling Technology	Cat# 9271, RRID:AB 329825	WB (1:1000)
Antibody	Anti-AKT (pan) (C67E7) (Rabbit monoclonal)	Cell Signaling Technology	Cat# 4691, RRID:AB_915783	WB (1:2000)
Antibody	Anti-phospho-p70S6 Kinase (Thr389) (108D2) (Rabbit monoclonal)	Cell Signaling Technology	Cat# 9234, RRID:AB_2269803	WB (1:2000)
Antibody	Anti-p70S6 Kinase (49D7) (Rabbit monoclonal)	Cell Signaling Technology	Cat# 2708, RRID:AB_390722	WB (1:2000)
Antibody	Anti-Pan-Actin (Rabbit polyclonal)	Cell Signaling Technology	Cat# 4968, RRID:AB_2313904	WB (1:2000)
Antibody	Anti-Rabbit Immunoglobulins/HRP (Goat polyclonal)	Dako, Agilent	Cat# P0448, RRID:AB_2617138	WB (1:2000)
Antibody	Anti-Mouse IgG (H + L), HRP (Rabbit polyclonal)	Thermo Fisher Scientific	Cat# 31457, RRID:AB_228439	WB (1:2000)
Antibody	Anti-MHC-I (Mouse monoclonal)	DSHB	Cat# BA-D5, RRID:AB_2235587	IF (1 µg/mL)
Antibody	Anti-MHC-IIA (Mouse monoclonal)	DSHB	Cat# SC-71, RRID:AB_2147165	IF (10 µg/mL)
Antibody	Anti-MHC-IIB (Mouse monoclonal)	DSHB	Cat# BF-F3, RRID:AB_2266724	IF (1 µg/mL)
Antibody	Anti-MHC-IIX (Mouse monoclonal)	DSHB	Cat# 6H1, RRID:AB_1157897	IF (1 µg/mL)
Antibody	Anti-embryonic myosin heavy chain (eMyHc) (Mouse monoclonal)	DSHB	Cat# F1.652, RRID:AB_528358	IF (20 µg/mL)
Antibody	Anti-Pax7 (Mouse monoclonal)	DSHB	Cat# PAX7, RRID:AB_2299243	IF (4 µg/mL)
Antibody	Anti-F4/80 (D4C8V) XP (Rabbit monoclonal)	Cell Signaling Technology	Cat# 30325, RRID:AB_2798990	IF (0.5 µg/mL)
Antibody	Anti-Myogenin (Mouse monoclonal)	DSHB	Cat# f5d, RRID:AB_2146602	IF (0.6 µg/mL)
Antibody	Anti-Ki67 (Rabbit monoclonal)	Cell Signaling Technology	Cat# 11882, RRID:AB_2687824	IF (1:200)
Antibody	Anti-Mouse Alexa Fluor 647 IgG_2b_ (Goat polyclonal)	Thermo Scientific	Cat# A-21242, RRID:AB_2535811	IF (5 µg/mL)
Antibody	Anti-Mouse Alexa Fluor 488 IgG_1_ (Goat polyclonal)	Thermo Scientific	Cat# A-21121, RRID:AB_2535764	IF (5 µg/mL)
Antibody	Anti-Mouse Alexa Fluor 647 IgM (Goat polyclonal)	Thermo Scientific	Cat# A21238, RRID:AB_1500930	IF (5 µg/mL)
Antibody	Anti-Mouse Alexa Fluor 488 IgM (Goat polyclonal)	Thermo Scientific	Cat# A-21042, RRID:AB_141357	IF (5 µg/mL)
Antibody	Anti-Mouse Alexa Fluor 488 IgG (H + L) (Goat polyclonal)	Thermo Scientific	Cat# A-11029, RRID:AB_2534088	IF (4–5 µg/mL)
Antibody	Anti-rabbit IgG (H + L), F(ab')2 Fragment Alexa Fluor 488 Conjugate (Goat polyclonal)	Cell Signaling Technology	Cat# 4412, RRID:AB_1904025	IF (5 µg/mL)
Sequence-based reagent	*Rps13*-F	This paper	PCR primers	CACGTGGCTGAAGTTGACG
Sequence-based reagent	*Rps13*-R	This paper	PCR primers	CAGGATTACACCTATCTGGGAGG
Sequence-based reagent	*Rpl27*-F	This paper	PCR primers	AGCCGTCATCGTGAAGAAC
Sequence-based reagent	*Rpl27*-R	This paper	PCR primers	GGGGATAGCGGTCAATTCC
Sequence-based reagent	*Tgfbr1*-F	This paper	PCR primers	CCTCGAGACAGGCCATTTGT
Sequence-based reagent	*Tgfbr1*-F	This paper	PCR primers	AGACGAAGCAGACTGGACCA
Sequence-based reagent	*Acvr1b*-F	This paper	PCR primers	TGCTGCGCCATGAAAACATC
Sequence-based reagent	*Acvr1b*-F	This paper	PCR primers	TGCCCACAATCTCCATATGCA
Sequence-based reagent	*Tgfb1*-F	This paper	PCR primers	GCTGACCCCCACTGATACG
Sequence-based reagent	*Tgfb1*-R	This paper	PCR primers	CCTGTATTCCGTCTCCTTGGTT
Sequence-based reagent	*Mstn*-F	This paper	PCR primers	GAGAATGGCCATGATCTTGCTG
Sequence-based reagent	*Mstn*-R	This paper	PCR primers	CTTCTAAAAAGGGATTCAGCCCATC
Sequence-based reagent	*Igf1ea*-F	This paper	PCR primers	GTGTTGCTTCCGGAGCTGTG
Sequence-based reagent	*Igf1ea*-R	This paper	PCR primers	CAATGTACTTCCTTCTGAGTC
Sequence-based reagent	*Hgf*-F	This paper	PCR primers	GATTATTGCCCTATTTCCCGTTGTG
Sequence-based reagent	*Hgf*-R	This paper	PCR primers	TGGCACAGGATATTACAGGATGG
Sequence-based reagent	*Igf1ec*-F	This paper	PCR primers	GGAGAAGGAAAGGAAGTACATTTG
Sequence-based reagent	*Igf1ec*-R	This paper	PCR primers	CCTGCTCCGTGGGAGGCT
Sequence-based reagent	*Vegfa*-F	This paper	PCR primers	CTGTAACGATGAAGCCCTGGAGTG
Sequence-based reagent	*Vegfa*-R	This paper	PCR primers	GGTGAGGTTTGATCCGCATGATCT
Sequence-based reagent	*Pax7*-F	This paper	PCR primers	TCCATCAAGCCAGGAGACA
Sequence-based reagent	*Pax7*-R	This paper	PCR primers	AGGAAGAAGTCCCACACAG
Sequence-based reagent	*Myod*-F	This paper	PCR primers	CATCCAGCCCGCTCCAAC
Sequence-based reagent	*Myod*-R	This paper	PCR primers	GGGCCGCTGTAATCCATCATGCC
Sequence-based reagent	*Myog*-F	This paper	PCR primers	CCCAACCCAGGAGATCATTT
Sequence-based reagent	*Myog*-R	This paper	PCR primers	GTCTGGGAAGGCAACAGACA
Sequence-based reagent	*Myh3*-F	This paper	PCR primers	CGCAGAATCGCAAGTCAATA
Sequence-based reagent	*Myh3*-R	This paper	PCR primers	CAGGAGGTCTTGCTCACTCC
Sequence-based reagent	*Id1*-F	This paper	PCR primers	ACCCTGAACGGCGAGATCA
Sequence-based reagent	*Id1*-R	This paper	PCR primers	TCGTCGGCTGGAACACAT
Sequence-based reagent	*Fgf2*-F	This paper	PCR primers	AAGCGGCTCTACTGCAAGAA
Sequence-based reagent	*Fgf2*-R	This paper	PCR primers	GTAACACACTTAGAAGCCAGCAG
Sequence-based reagent	*Ccn2*-F	This paper	PCR primers	CCACCCGAGTTACCAATGAC
Sequence-based reagent	*Ccn2*-R	This paper	PCR primers	GCTTGGCGATTTTAGGTGTC
Sequence-based reagent	*Col1a1*-F	This paper	PCR primers	ATGTTCAGCTTTGTGGACCT
Sequence-based reagent	*Col1a1*-R	This paper	PCR primers	CAGCTGACTTCAGGGATGT
Sequence-based reagent	*Col3a1*-F	This paper	PCR primers	AAGGACATCGAGGATTCCCTG
Sequence-based reagent	*Col3a1*-R	This paper	PCR primers	AGCCCTCAGATCCTCTTTCAC
Sequence-based reagent	*Cd68*-F	This paper	PCR primers	TCCCAACAAAACCAAGGTCCA
Sequence-based reagent	*Cd68*-R	This paper	PCR primers	GGCTCTGATGTAGGTCCTGTTT
Sequence-based reagent	*Cd163-*F	This paper	PCR primers	CGGCCCCATGAAGAGGTATC
Sequence-based reagent	*Cd163*-R	This paper	PCR primers	GACGGTTGACCCAGTTGTTG
Sequence-based reagent	*Il1b*-F	This paper	PCR primers	GCCACCTTTTGACAGTGATG
Sequence-based reagent	*Il1b*-R	This paper	PCR primers	CTTCTCCACAGCCACAATGA
Sequence-based reagent	*Il6*-F	This paper	PCR primers	GGAAATGAGAAAAGAGTTGTGC
Sequence-based reagent	*Il6*-R	This paper	PCR primers	GTACTCCAGAAGACCAGAGGA
Sequence-based reagent	*Fbxo32*-F	This paper	PCR primers	AGACTGGACTTCTCGACTGC
Sequence-based reagent	*Fbxo32*-R	This paper	PCR primers	TCAGCTCCAACAACAGCCTTACT
Sequence-based reagent	*Trim63-*F	This paper	PCR primers	CGTCCAGAGCGTGTGTCTCACTC
Sequence-based reagent	*Trim63-*R	This paper	PCR primers	GGGCTACCTTCCTCTCAAGTGC
Sequence-based reagent	*Tcf4-*F	This paper	PCR primers	GGAAAGCCCTAGCTTCGATCT
Sequence-based reagent	*Tcf4-*R	This paper	PCR primers	GGAGCCCACAGGAGTTGAA
Sequence-based reagent	*Pdgfra-*F	This paper	PCR primers	ACTTTTCACTCCGGGTATCGG
Sequence-based reagent	*Pdgfra*-R	This paper	PCR primers	CCCATAGCTCCTGAGACCTTC
Commercial assay or kit	RiboPure RNA Purification Kit	Thermo Fisher Scientific	AM1924	
Commercial assay or kit	SuperScript VILO Mastermix	Thermo Fisher Scientific	12023679	

### Animal housing and welfare

The HSA-Cre transgenic mouse line (McCarthy et al., 2012b) was obtained from Jackson Laboratory, Bar Harbor, ME,USA (stock number # 025750), the *Acvr1b^fl/fl^* mouse line (Ripoche et al., 2013) was obtained from Philippe Bertolino (Cancer Research Center of Lyon, French Institute of Health and Medical Research) and the *Tgfbr1^fl/fl^* mouse line (Larsson et al., 2001) was provided by Peter ten Dijke (Leiden University Medical Center). All mouse lines were of a C57BL/6 background. Mouse lines were cross-bred in house to obtain mouse lines HSA-Cre:*Acvr1b^fl/fl^* (*Acvr1b* CKO), HSA-Cre:*Tgfbr1^fl/fl^* (*Tgfbr1* CKO) and HSA-Cre:*Acvr1b^fl/fl^:Tgfbr1^fl/fl^* (*Acvr1b:Tgfbr1* CKO). Animals were housed in a controlled 12 hr light-dark cycle (light on 6:00-18:00 GMT +1 hr) with a temperature of 21°C ± 1°C and a humidity between 40% and 70%. Food (Teklad, Envigo, Horst, The Netherlands) and water were available at libitum. All experiments were performed according to the national guidelines approved by the Central Committee for Animal Experiments (CCD) (AVD112002017862) and the Institute of Animal Welfare (IvD) of the Vrije Universiteit Amsterdam.

### Genotyping

In these mouse models, skeletal myofibre-specific Cre expression is driven by the *ACTA1* promoter (HSA-Cre) which can be activated by TMX, resulting in the deletion of targeted exon of 5 and 6 of *Acvr1b* and exon 3 of *Tgfbr1* resulting in a targeted knockout of the gene. Genotyping for the *HSA-Cre*, *Acvr1b^fl/fl^* and *Tgfbr1^fl/fl^* genes was performed by isolating DNA from ear biopsies of offspring and PCR was performed in a 2720 thermal cycler (Applied Biosystems, Foster City, CA, USA). PCR master mix per sample was prepared by mixing 0.2 μl of AmpliTaq Gold DNA polymerase, 2.5 μl of gold buffer, 1.5 μl of MgCl_2_ (Thermo Fisher Scientific, 4311806, Waltham, MA, USA), 0.5 μl of dNTPs (100 mM diluted 10×, Invitrogen 10297018, Carlsbad, CA, USA), 1 μl of each primer diluted in DNAse/RNAse free water to obtain a volume of 23 μl Master mix per sample. Two μl DNA was added per sample. The following PCR programs were used: for *HSA-Cre* and *Acvr1b^fl/fl^*: 94 °C for 5 min, followed by a 35 × cycle of 94 °C for 30 s, 58 °C for 30 s and 72 °C for 10 min, finishing with 72 °C for 10 min and cooled down to 4 °C. PCR program for *Tgfbr1^fl/fl^*: 94 °C for 4 min, followed by a 35 × cycle of 94 °C for 30 s, 50 °C for 45 s and 72 °C for 1 min, finishing with 72 °C for 5 min and cooled down to 4 °C. Amplified DNA was mixed with 5 μl loading buffer and samples were loaded in a 4% agarose gel using SYBR safe DNA gel staining 1000 × concentrate (Thermo Fisher Scientific s33102, Waltham, MA, USA), DNA was separated by electrophoresis (25 minutes, 75 V) and gel image was taken using an Image Quant LAS 500 chemo luminescence CCD camera (GE healthcare, life sciences, Chicago, IL, USA).

Primer sequences: *HSA-Cre* gene: Forward, 5′- GCATGGTGGAGATCTTTGA-3′ (McCarthy et al., 2012b) and Reverse, 5′-CGACCGGCAAACGGACAGAAGC-’3 (McCarthy et al., 2012a). *Acvr1b^fl/fl^* gene: *Acvr1b In4*, 5’-CAGTGGTTAAGAACACTGGC-3’, *Acvr1b In5*, 5’- GTAGTGTTATGTGTTATTGCC –3’ and *Acvr1b In6*, 5’GAGCAAGAGTTTCTCTATGTAG-3’ (Ripoche et al., 2013). *Tgfbr1^fl/fl^* gene: Forward, 5’- CCTGCAGTAAACTTGGAATAAGAAG-’3, Reverse, 5’- GACCATCAGCTGTCAGTACCC-3’ (Protocol 19216: Standard PCR Assay - Tgfbr1 <tm1.1Karl>, Jackson Laboratory, Bar Harbor, ME,USA).

### Cardiotoxin-induced injury assay

Animals of each genotype were assigned to each timepoint randomLy to ensure that groups were on average the same age at the time of the first TMX injection. Littermates were assigned to different timepoints. Six weeks old *Acvr1b* CKO, *Tgfbr1* CKO, *Acvr1b:Tgfbr1* CKO and *HSA-Cre* Cre^+^ male mice were injected intraperitoneally with 100 mg/kg/day tamoxifen (Sigma-Aldrich, T5648, Saint-Louis, MO, USA) in sunflower oil (10 mg/mL) for 5 consecutive days. Five weeks post TMX injections, mice were injected intramuscularly in the TA muscles of both hind limbs with 20 µL CTX from Naja pallida (Latoxan Laboratory, L8102, Portes les Valence, France) in phosphate buffered saline (PBS) (10 µM). CTX was slowly injected (1 µL/s) into mid muscle belly using a Hamilton syringe with attached 34 G needle inserted in a 15–25° angle, 2–3 mm deep. Mice were shortly anesthetised using isoflurane 1.5–3% on a warm blanket during the injections. Mice were divided into three groups: mice that were sacrificed 2 days or 4 days post injury and mice with no CTX injection, that were sacrificed at day 0, which functioned as a baseline control. Mice were sacrificed by cervical dislocation and TA muscles were isolated and frozen in isopentane cooled in liquid nitrogen. Each subgroup contained 5–8 mice.

### RNA isolation and reverse transcription

Whole TA muscle was used for RNA isolation. 50 mg cryopreserved TA muscle was homogenised (Potter S 8533024, B. BRAUN) in 700 µL TRI reagent (Invitrogen, 11312940, Carlsbad, CA, USA) and incubated at room temperature (RT) for 5 min. Samples were centrifuged for 10 min (4 °C, 12,000 g). Supernatant was transferred to a new tube and 70 µL bromochloropropane (Sigma-Aldrich, B9673, Saint Louis, MO, USA) was added. Lysates were inverted and incubated at RT for 5 min and centrifuged (4 °C, 12,000 g, 10 min). RNA containing supernatant was transferred to a new centrifuge tube and washed with 100% ethanol 2:1. RNA was further isolated using the RiboPure RNA purification kit (Thermo Fisher Scientific, AM1924, Waltham, MA, USA). Then, 500 ng RNA and 4 µL SuperScript VILO Mastermix (Invitrogen, 12023679, Carlsbad, CA, USA) were diluted to 20 µL in RNAse free water and reverse transcription was performed in a 2720 thermal cycler (Applied Biosystems, Foster City, CA, USA), using the following program: 10 min at 25 °C, 60 min at 42 °C and 5 min at 85 °C. cDNA was diluted 10 × in RNAse-free water.

### Quantitative real-time PCR

Five μl of PowerUp SYBR Green master mix (Applied Biosystems, A25742, Foster City, CA, USA), 3 μL of primer mix and 2 μl of cDNA were added in duplo in a 96 wells plate. The program ran on the Quant Studio 3 real time PCR (Applied Biosystems, Foster City, CA, USA) was 2 min at 50°C, 2 min at 95°C, 40 × 1 s at 95°C and 30 s at 60°C, 15 s at 95°C, 1 min at 60°C and 15 s at 95°C. Geometric mean of reference genes ribosomal protein S13 (*Rps13*) and ribosomal protein L27 (*Rpl27*) was used to correct for cDNA input. The efficiency of all primers sequences (Key Resources Table) was >98%.

### Western blot

Fifty 20 μm cross-sections of TA and EDL muscles were obtained using a cryostat microtome (Microm HM550, Adamas Instruments, Rhenen, The Netherlands). GM tissue and whole EDL muscles were lysed (Potter S 8533024, B. BRAUN) in RIPA buffer (Sigma-Aldrich, R0278, Saint Louis, MO, USA) containing 1 tablet of protease inhibitor (Sigma-Aldrich, 11836153001) and 1 tablet of phosStop (Sigma-Aldrich, 04906837001) per 10 mL. The total protein concentration in the lysates was determined using a Pierce BCA Protein Assay kit (Thermo Scientific, 23225). A 4–20% Mini-PROTEAN TGX Precast Protein Gels was used. Fifteen µL sample mix containing 12 µg total protein and 4 µL sample buffer (4 × Laemmli Sample Buffer, Bio-Rad, 1610747) with 10% mercaptoethanol (Bio-Rad, 1610710) was heated to 95 °C for 5 min, cooled on ice and loaded onto the gel. After electrophoresis, proteins were transferred onto a polyvinylidene fluoride (PVDF) membrane (GE Healthcare, 15269894) for blotting at 80 V for 60 min. The membrane was incubated for 1 hr at RT in 2% enhanced chemiluminiscence (ECL) prime blocking agent (GE Healthcare, RPN418). Membranes were incubated overnight at 4 °C in blocking buffer (4% bovine serum albumin (BSA) in tris-buffered saline with 0.1% Tween 20 detergent (TBST)) with primary antibody at a dilution of 1:500 for anti-phospho-Smad2 (Ser465/467) (138D4), anti-phospho-Smad3 (Ser423/425) (C25A9) and for anti-Smad2/3, 1:000 for anti-phospho-AKT (Ser473), 1:2000 for anti-AKT (pan) (C67E7), anti-phospho-p70S6 Kinase (Thr389) (108D2), anti-p70S6 Kinase (49D7) and anti-pan-Actin. Incubation with secondary antibody, anti-rabbit IgG HRP conjugated or anti-mouse IgG, IgM (H + L), HRP conjugatd at a dilution of 1:2000 was done for 1 hr at RT in blocking buffer and detection was done using ECL detection kit (RPN2235, GE Healthcare, USA). Images were taken by the ImageQuant LAS500 (GE healthcare, life sciences, USA) and relative intensity of protein bands was quantified using ImageJ (Schneider et al., 2012). Pan-Actin was used as a loading control.

### Tissue cross-sectioning for histological analysis

For histological analysis, 10-μm-thick cross-sections of TA or EDL muscles were obtained using a cryostat microtome (Microm HM550, Adamas Instruments, Rhenen, The Netherlands), mounted on microscope slides (super frost plus, Thermo Scientific, J1800AMNZ, Landsmeer, The Netherlands) and stored at –80 °C for further analyses. In addition, for TA muscle, 10-μm-thick longitudinal sections were obtained to measure the myonuclear length.

### Histochemistry staining of H&E and Sirius Red staining

For H&E staining, slides with muscle sections were air dried for 10 min. Dried slides were then stained in Hematoxylin for 2 min, then washed under tap water for 15 min and further stained in Eosin for 1 min. Immediately after Eosin staining, slides were rinsed 2 × in 100% alcohol for 10 s and 2 × in xylene for 10 min. Stained sections were mounted in Entellan (Merck, 107960, Darmstadt, Germany), covered with coverslip and dried overnight before analysis.

For Sirius Red staining, sections were air dried for 10 min at RT, then they were fixed in acetone at –20 °C for 10 min and subsequently fixed in saturated Bouin for 30 min at RT. Samples were stained by Sirius Red for 30 min and washed in 0.01 M hydrochloric acid. Then, sections were rapidly dehydrated in 100% ethanol and xylene twice. Stained sections were mounted in Entellan, covered with coverslip and dried overnight before analysis (Bruin et al., 2014).

Images were captured on a Zeiss Axioskop microscope (Carl Zeiss BV, Breda The Netherlands) with Basler camera (Basler AG, Ahrensburg, Germany) using Manual WSI scanner software (Microvisioneer) for collecting whole scan images. Regeneration area percentage at day 0 was referred to as the area of myofibres with central nuclei divided by total the area of a whole muscle cross-section. Morphometry was performed using ImageJ software (Schneider et al., 2012).

### Immunofluorescence staining

Microscope slides with muscle sections were air dried for 10 min. For Pax7 staining, sections were fixed with 4% paraformaldehyde (PFA) at RT for 10 min and washed in PBS-T (0.05% Tween 20). Sections were blocked at RT with 10% normal goat serum (NGS) (Thermo Fisher Scientific, 50,062Z, Waltham, MA, USA) (60 min for myosin heavy chain (MyHC), eMyHC, F4/80 and Myogenin staining, or 30 min for Pax7 staining) or 5% NGS with 0.3% Triton-X100 for 60 min for Ki67/Pax7 staining. Sections for Pax7 staining were subsequently blocked in 1% BSA in PBS for 30 min. Sections were incubated in primary antibodies (Key Resources Table) in 10% NGS (for MyHC, eMyHC or Myogenin) or in 0.1% BSA (Pax7, F4/80 or Ki67/Pax7) at RT for 60 min or overnight. Then sections were incubated in secondary antibodies (Key Resources Table) in 10% NGS for MyHC and eMyHC staining, or in 0.1% BSA for Pax7 or F4/80 staining at RT in the dark for 60 min. After this, sections were incubated in 1:50 diluted wheat germ agglutinin (WGA) (Fisher Scientific, 11590816, Pittsburgh, PA, USA) in PBS at RT for 20 min. Finally, slides were carefully dried and mounted with Vectashield hardset mounting medium with 4‘,6-diamidino-2-phenylindole (DAPI) (Brunschwig, H1500, Amsterdam, The Netherlands). Slides were dried overnight at 4 °C.

### Immunofluorescence microscopy and analysis

Images of all immunofluorescence assays were captured using a fluorescent microscope (Zeiss Axiovert 200 M, Hyland Scientific, Stanwood, WA, USA) with a PCO SensiCam camera (PCO, Kelheim, Germany) using the program Slidebook 5.0 (Intelligent Imaging Innovations, Göttingen, Germany). The images were analysed using ImageJ (Schneider et al., 2012). Individual images were taken across the entire cross-section and assembled into a composite panoramic image. For TA, the inner part of the muscle tissue was referred to as high oxidative region and the outer part was referred to as low oxidative region. For myofibre type analysis, 250 myofibres within each part were characterised in TA. Hybrid myofibre fluorescence was assessed by myofibres stained by double colours and pure myofibres were determined by single colour staining (Bloemberg and Quadrilatero, 2012). To determine CSA per myofibre type, CSA was measured of 30 myofibres per type, or as many as were present within the tissue per TA. For EDL, myofibre type and CSA of all myofibres within the muscle were determined by SMASH (Smith and Barton, 2014). Within images stained for Pax7, the number of Pax7^+^ nuclei and the number of myofibres were determined in the low oxidative region of TA from about 200 myofibres (5 fields per specimen). SCs were defined as Pax7^+^ cells that were located between plasma and basal lamina of myofibre (Lindstrom and Thornell, 2009). Within muscle sections images stained for F4/80, the density of macrophages in TA at day 0 was determined in 10 randomly selected locations and in at least 3 locations within the injured region at day 2 and 4 after injury. Macrophages were defined as F4/80^+^ cells that were located in the interstitial region of the myofibres at day 0. To measure the number of myonuclei per type IIB myofibre, 100 type IIB myofibres in the low oxidative region were taken into account and nuclei were considered as myonuclei when they were located within the cytoplasm below the basal lamina. To determine myonuclear length, images were taken at ×10 magnification using a fluorescent microscope. Mean myonuclear length was determined as the average value of 30 nuclei. For regeneration analysis, myofibre CSA measurement was performed by outlining 50 eMyHC^+^ myofibres from three randomly chosen fields within the regenerating area in muscle cross-sections of day 4. RI was defined as the number of nuclei within eMyHC^+^ myofibres divided by the number of eMyHC^+^ myofibres, all eMyHC^+^ myofibres in three randomly chosen fields (1.38 mm^2^ per field) within the regenerating area were included in the analysis. The densities of Ki67^+^, Pax7^+^, Ki67^+^/Pax7^+^ and Myogenin^+^ cells in TA were determined by counting the number of cells per mm^2^ of muscle CSA. Ten images in low oxidative region of TA on days 0, 2, and 4 were randomly selected. All analyses were performed at ×20 magnification.

### SDH assay

Succinate dehydrogenase activity was quantified according to van der Laarse et al., 1989. Breiefly, freshly muscle cross-sections (10 µm thick) were air dried for 15 min at RT. Sections were incubated in prewarmed SDH medium (37.5 mM NaH_2_PO_4_.H_2_O, 37.5 mM Na_2_HPO_4_.2H_2_O, added acid to pH 7.6, 75 mM sodium succinate, 5 mM NaN_3_, 0.5 g/L tetranitroblue tetrazolium (TNBT)) for 10 min at 37 °C. Sections were rinsed 3 seconds in 0.01 M hydrochloric acid. Then sections were rinsed for 1 min twice in ultrapure water. Finally, sections were mounted in glycerine gelatin (Merck, 48723, Darmstadt, Germany) with coverslips and dried overnight before analysis. Images were captured by a Leica DMRB microscope (Wetzlar, Germany) with calibrated grey filters and a CCD camera (Sony XC77CE, Towada, Japan) connected to a LG-3 frame grabber (Scion, Frederick, MD, United States). The absorbances of the SDH-reaction product in the myofibre cross-sections were determined at 660 nm using a calibrated microdensitometer and ImageJ (Schneider et al., 2012). SDH activity (ΔA_660_∙µm^–1^∙s^–1^) was quantified at 5 locations in low oxidative region at ×10 magnification by measuring the rate of absorbance per section thickness per second (ΔA_660_/(10 µm∙600 s)) after subtracting background activity. The integrated SDH activity (ΔA_660_∙µm∙s^–1^) was defined as SDH activity ×myofibre CSA. Absorbance was measured in a total of 50 myofibres per TA.

### Statistical analysis

Graphs were made in Prism version 8 (GraphPad software, San Diego, CA, USA). All data were presented as mean + standard error of the mean (SEM). Statistical analysis was performed in SPSS version 26 (IBM, Amsterdam, The Netherlands). Sample size was determined a priori, based on previously published in vivo research on TGF-β and myostatin. Statistical significance for multiple comparisons was determined by two-way analysis of variance (ANOVA), three-way ANOVA or independent t-test. Significance was set at p < 0.05. Data normality was tested with a Shapiro-Wilk test (p < 0.05). Homogeneity of variance was tested with a Levene’s test (p < 0.01). If necessary, data were square or log transformed. Post hoc Bonferroni or Games-Howell corrections were performed. If normality was violated a Kruskall Wallis test was performed.

## Data Availability

All data generated or analysed during this study are included in the manuscript and supporting file; Source Data files have been provided for Figures 1 to 5. The following dataset was generated: HillegeMMG ShiA GalliRA WuG BertolinoP HoogaarsWMH JaspersRT 2022Data from: Lack of Tgfbr1 and Acvr1b synergistically stimulates myofibre hypertrophy and accelerates muscle regenerationDryad Digital Repository10.5061/dryad.jh9w0vtd5PMC900518735323108

## Additional files

Transparent reporting form
