# Supplementary figures and images for: Lack of Tgfbr1 and Acvr1b synergistically stimulates myofibre hypertrophy and accelerates muscle regeneration
Source: eLife. 2022 Mar 24;11:e77610. doi: 10.7554/eLife.77610 (PMC9005187; doi:10.7554/eLife.77610)

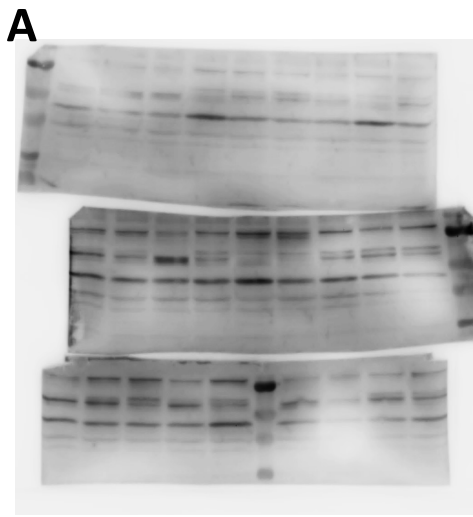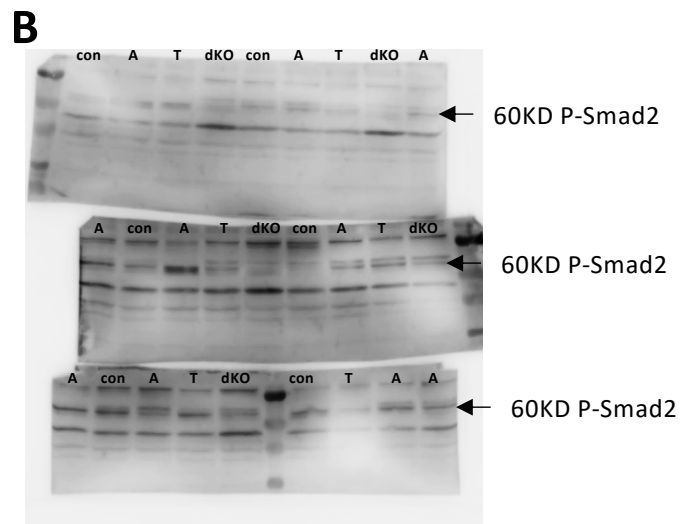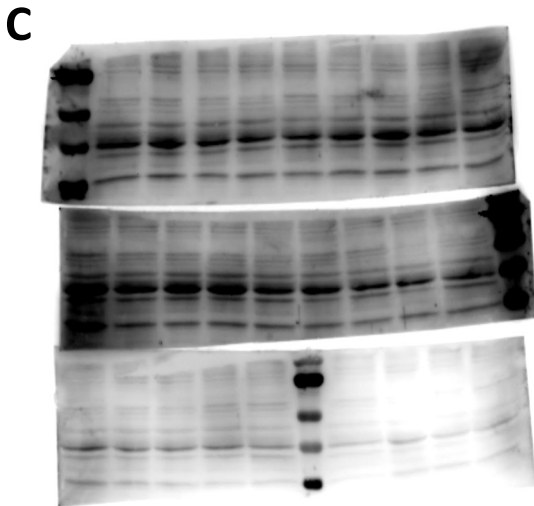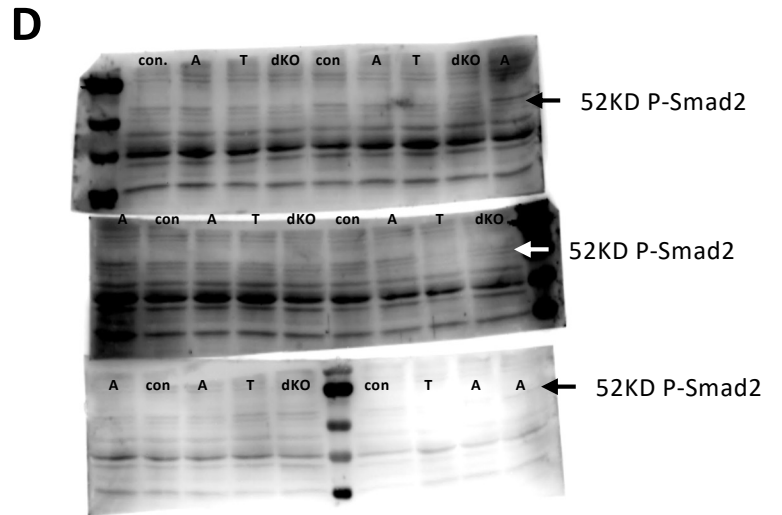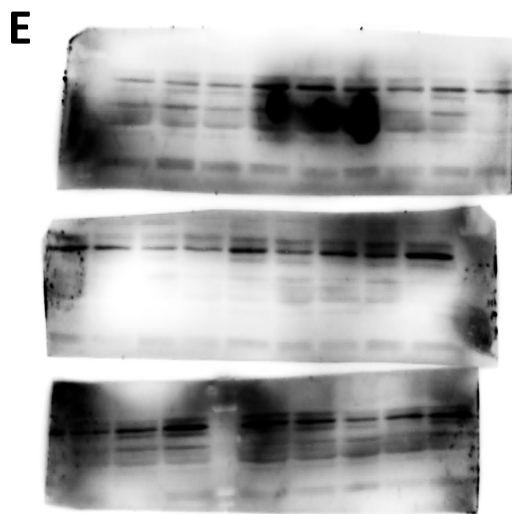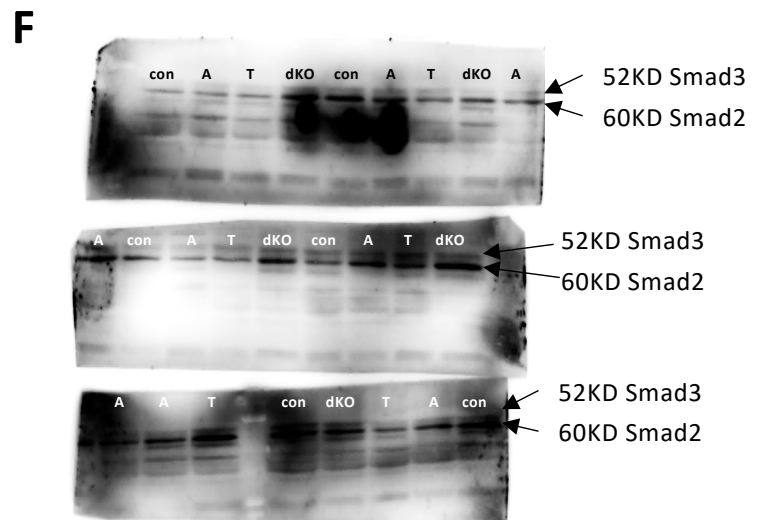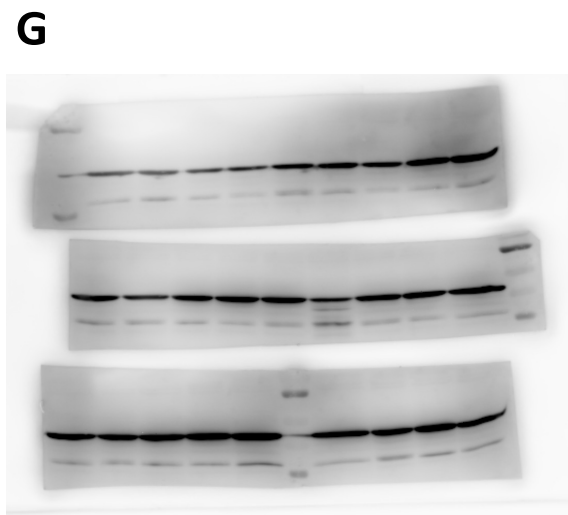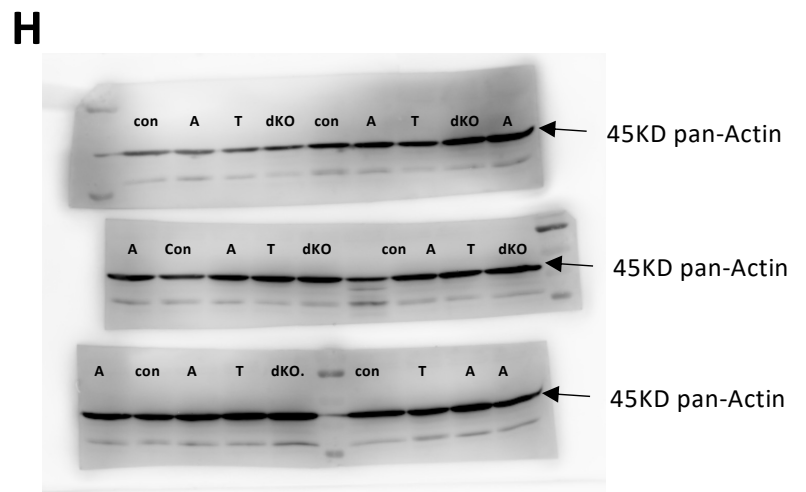

Supplement: Figure 3—source data 2. — Labelled images of (B) P-Smad2, (D) P-Smad3 and (F) Smad2/3 and (H) pan-Actin of tibialis anterior (TA) muscle in groups of control (con), Acvr1b CKO (A), Tgfbr1 CKO (T) and Acvr1b: Tgfbr1 CKO (dKO). [file elife-77610-fig3-data2.pdf]

**A**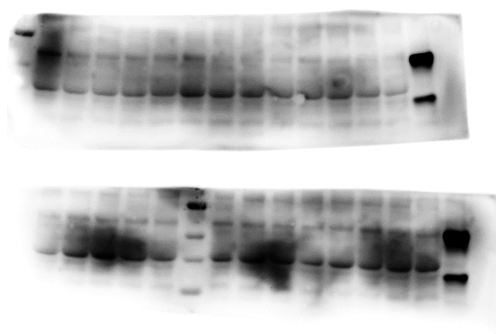**B**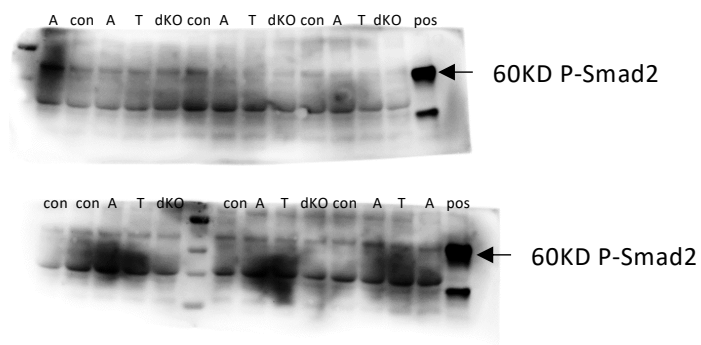**C**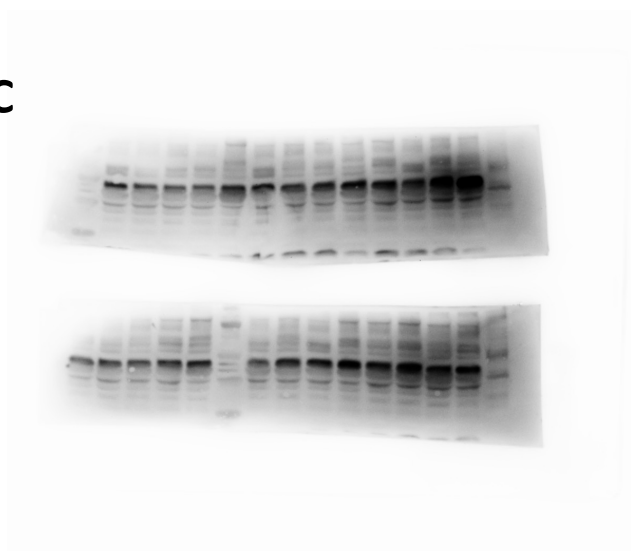**D**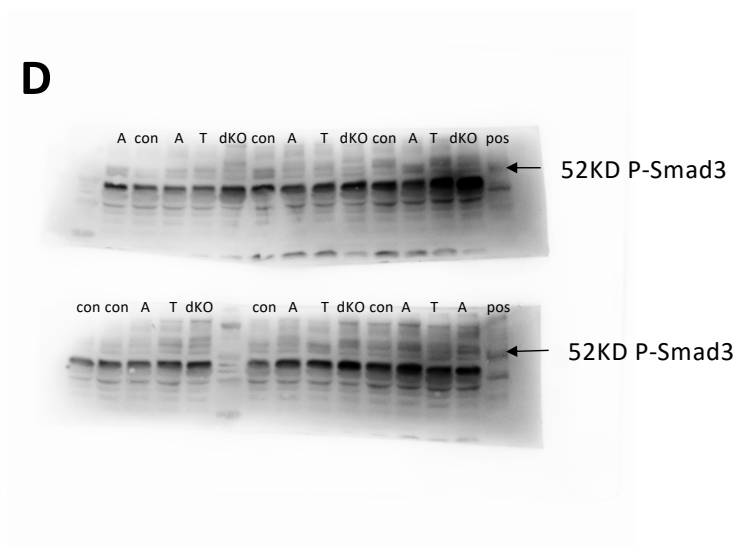**E**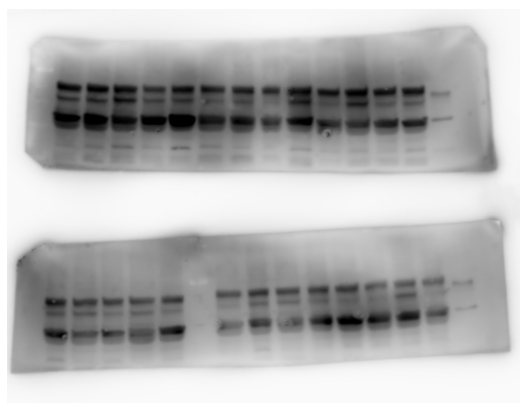**F**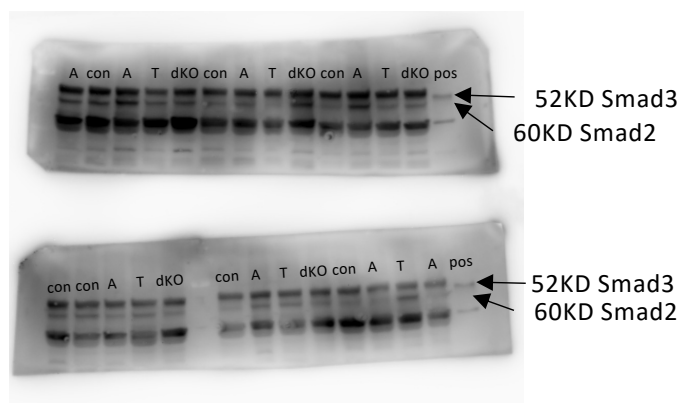**G**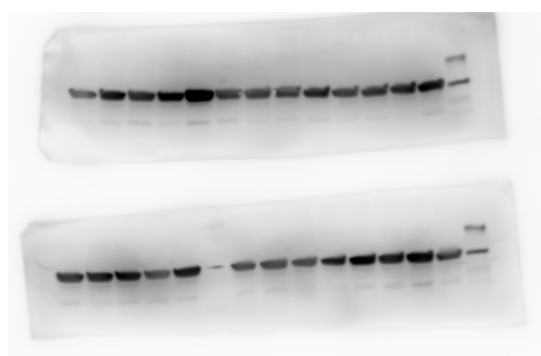**H**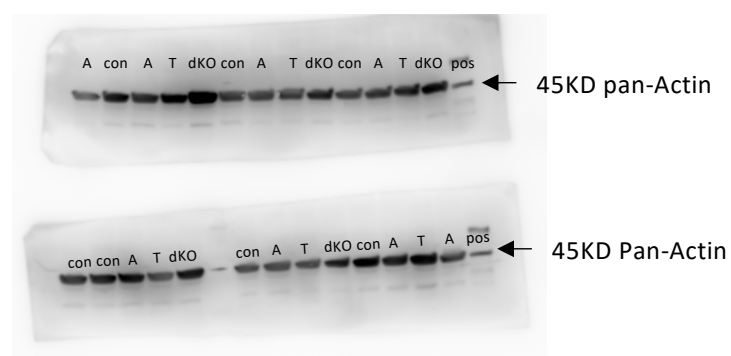

Supplement: Figure 3—source data 3. — Labelled images of (B) P-Smad2, (D) P-Smad3, and (F) Smad2/3 and (H) pan-Actin of extensor digitorum longus muscle (EDL) in groups of control (con), Acvr1b CKO (A), Tgfbr1 CKO (T), Acvr1b: Tgfbr1 CKO (dKO) and positive control sample (pos). [file elife-77610-fig3-data3.pdf]

**A**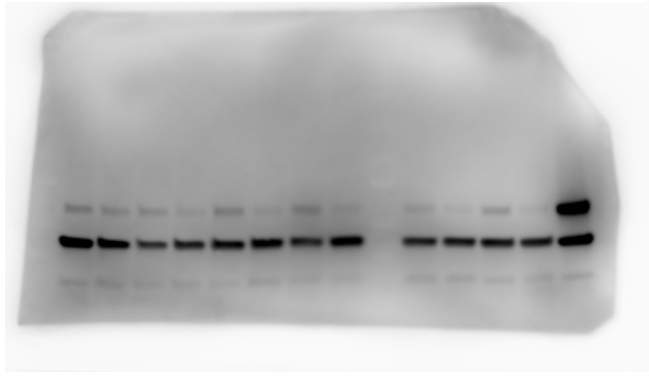**B**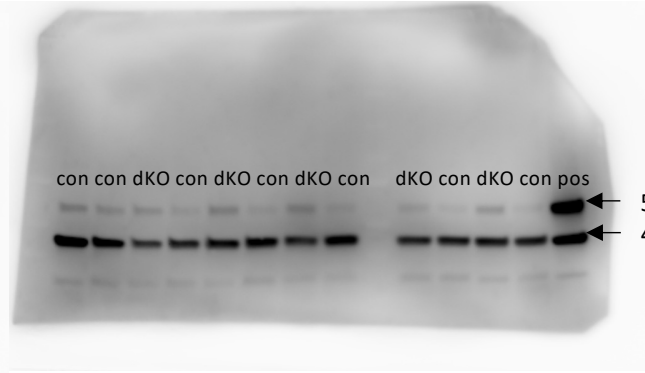**C**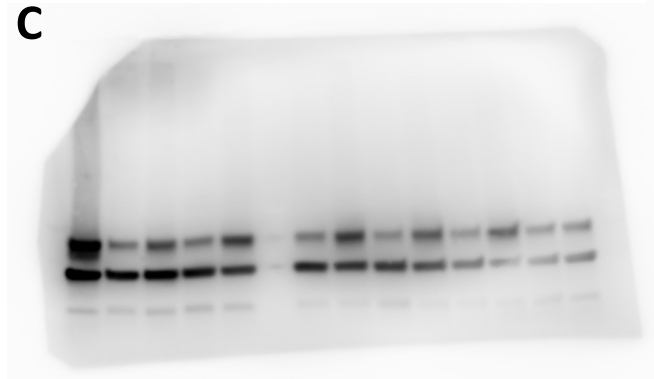**D**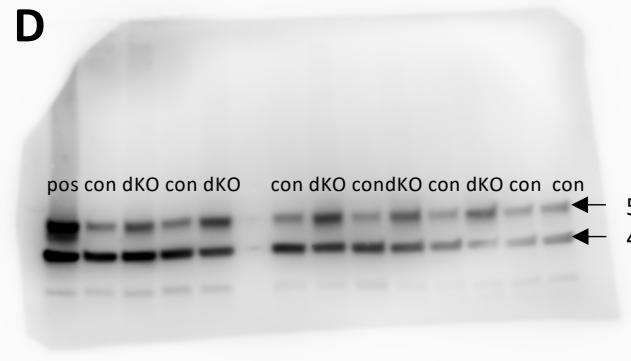**E**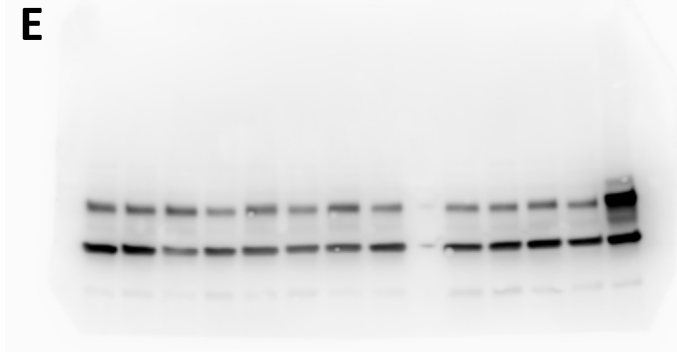**F**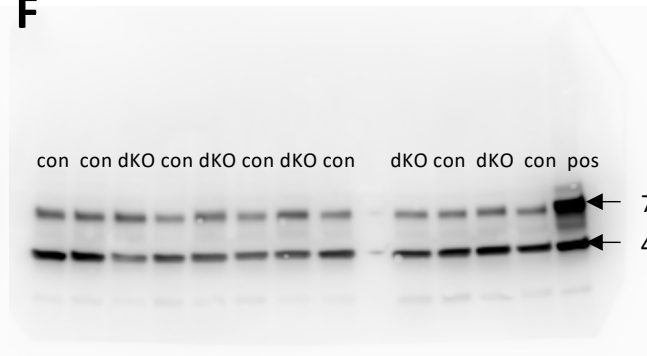**G**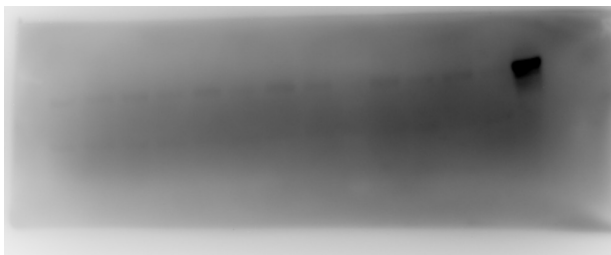**H**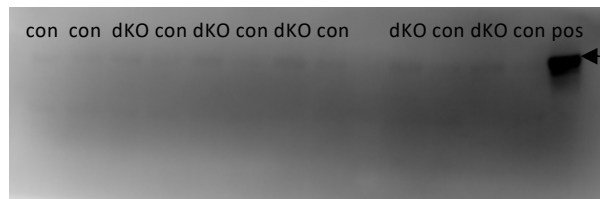**I**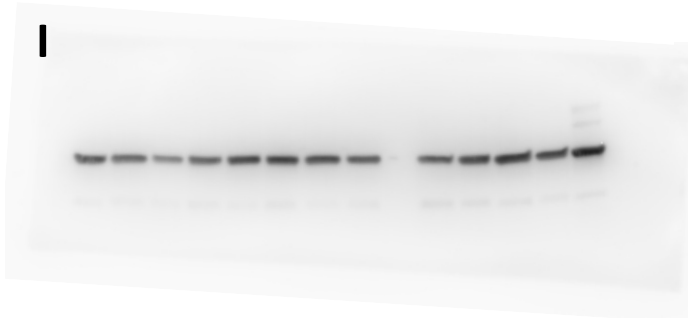**J**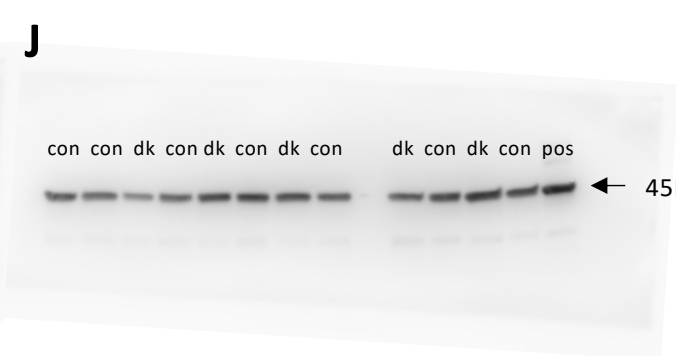

Supplement: Figure 3—source data 4. — Labelled images of (B) P-AKT, (D) AKT and (F) P-p70s6k, (H) p70s6k and (I) pan-Actin of TA in groups of control (con), Acvr1b: Tgfbr1 CKO (dKO) and positive control sample (pos). [file elife-77610-fig3-data4.pdf]
