# Supplementary material for: Lack of Tgfbr1 and Acvr1b synergistically stimulates myofibre hypertrophy and accelerates muscle regeneration
Source: eLife. 2022 Mar 24;11:e77610. doi: 10.7554/eLife.77610 (PMC9005187; doi:10.7554/eLife.77610)
Supplement: Peer review history

# Editor report: Editor's evaluation

**DOI:** 10.7554/eLife.77610.sa0

**Authors:** Christopher L-H Huang

**Affiliations:** https://ror.org/013meh722 University of Cambridge United Kingdom

This paper demonstrates that inhibition of TGF-β signalling by simultaneous muscle-specific knockout of TGF-β type I receptors. In particular Tgfbr1 and Acvr1b simultaneously, induces substantial hypertrophy, while such effects do not occur by single receptor knockout. These findings implicate these factors in a synergistic involvement in regulating myofibre size, regeneration and collagen deposition.

# Decision letter

**DOI:** 10.7554/eLife.77610.sa1

**Affiliations:** https://ror.org/013meh722 University of Cambridge United Kingdom; https://ror.org/02en5vm52 INSERM - CNRS - Université Pierre et Marie Curie France

In the interests of transparency, eLife publishes the most substantive revision requests and the accompanying author responses.

**Decision letter after peer review:**

Thank you for choosing to send your work, "Lack of *Tgfbr1* and *Acvr1b* synergistically stimulates myofibre hypertrophy and accelerates muscle regeneration", for consideration at *eLife*. Your submission has been assessed by a Senior Editor in consultation with a member of the Board of Reviewing Editors. Although the work is of interest, we regret to inform you that the findings at this stage are too preliminary for further consideration at *eLife*.

Specifically, we agree that the generation of muscle specific and inducible knockout mice for ALK4 and ALK5 is of importance for both translational aspects of muscle diseases and conceptual advances in the field of adult myogenesis. Yet, this work requires additional data to support the central claims of the paper. Due to prior literature in the field, specifically the effect of TGF-β pathways on myofiber size, the state of Phospho-Smad2/3 in vivo must be evaluated. Quantification of myofiber size will have to be more accurately measured (reviewer #3). Lastly, the study needs to clearly identify the cell types affected by the myofiber-specific KO during regeneration induced by acute tissue damage (reviewer #2). We would consider a revised version of the paper that include these additional aspects.

Essential revisions:

Due to prior literature in the field, specifically the effect of TGF-β pathways on myofiber size, the state of Phospho-Smad2/3 in vivo must be evaluated. Quantification of myofiber size will have to be more accurately measured (reviewer #3). Lastly, the study needs to clearly identify the cell types affected by the myofiber-specific KO during regeneration induced by acute tissue damage (reviewer #2). We would consider a revised version of the paper that include these additional aspects.

*Reviewer #1:*

TGF-β family growth factors whose signaling is dependent upon type I receptor TFGbr1 and Acvr1b phosphorylation are of widespread importance in cell signaling. This paper focusses on their modification in skeletal myocytes and their atrophic actions and fibrotic change. Such Tgf downregulatory effects have potential translational importance in muscle wasting disease of wide clinical importance. The paper fulfils its defined objectives. The experiments lead to a straightforward emergence of its conclusion bearing on muscle atrophy and recovery from injury.

Its strengths include clearcut demonstration leading to a clear narrative from experimental platform to scientific outcomes of (a) a straightforward downregulation of the target receptors in the knockout experimental platform. (b) a clearcut demonstration of a consequent IIB cardiomyocyte hypertrophic phenotype correlated with a succinate dehydrogenase downregulation compared to fl- attributed to (c) reciprocal positive and negative effects of Akt/mTOR/p70S6K and Murf-1 e signalling. (d) The findings were substantiated in an in vivo injury system further implicating extracellular matrix deposition. Thus, combined knockout of these TFGbr1 and Acvr1b receptors increases the number of satellite cells and improves regeneration post cardiotoxin-induced injury by stimulation of myogenic gene expression resulting in improved myogenesis.

Its weaknesses should prompt future physiological studies of a recovery of muscle function in terms of its triggering and tension generation capacity.

# Reply: Author response

**DOI:** 10.7554/eLife.77610.sa2

Essential revisions:

Quantification of myofiber size will have to be more accurately measured (reviewer #3).

We agree that quantification of all myofibres in a cross-section of a muscle is most reliable to measure myofibre cross-section area (CSA). However, it is difficult for the large tibialis anterior muscle to measure the size for all myofibres per myofibre type as there are also hybrid myofibers that express two or more myosin heavy chain isoforms. For TA muscle, we determined for each myofibre type the mean CSA based on the average CSA of 30 randomly selected myofibres. This was done as we had shown previously by measuring the size of all myofibres within EDL muscle (please see below) that an average value of 30 muscle fibres would give a sufficient accuracy [1]. The reviewers suggested we verify whether the effects of TGF-β type I receptors knockout apply exclusively to the TA muscle. To test this, we now have made cryo-sections for the EDL muscles and stained these for myosin heavy chain and quantified myofibre CSA for each myosin type based on all EDL myofibres within a cross-section (the myofibre numbers varied from 500-900 between individual muscles). The effects of Acvr1b^fl/fl^ and/or Tgfbr1^fl/fl^ were fairly similar and in the same order of magnitude as those observed for the TA muscle.

To confirm that the average values of TA myofibre CSA based on 30 myofibers was sufficient to obtain a valid and reliable estimate, we calculated for EDL muscles how the mean myofibre CSA would change when a lower number of myofibres was analysed rather than the total number within a cross-section. Comparison of the mean myofibre CSA from 30 myofibres yielded a mean myofibre CSA for the groups being less than 2% different from the mean value based on the total number of myofibers. These data confirmed our previous observation that the estimate for CSA based on 30 myofibres is reliable.

The data of the EDL muscle are now added to the manuscript and described in the methods and Results sections:

Results section:

“In contrast to observations in TA, CSA of type IIA and type IIX myofibres in EDL muscle of Acvr1b^fl/fl^:Tgfbr1^fl/fl^ mice were increased by 1.6-fold and 1.5-fold compared to those of control mice, respectively. However, similar to TA muscle, CSA of type IIB myofibres of Acvr1b^fl/fl^:Tgfbr1^fl/fl^ mice was increased most substantially compared to that of control mice (1.7-fold) (Figure 1F).”

Methods section:

“For EDL, myofibre type and CSA of all myofibres within the muscle were determined by SMASH [2].”

Figures: Figure 1 F is added

Due to prior literature in the field, specifically the effect of TGF-β pathways on myofiber size, the state of Phospho-Smad2/3 in vivo must be evaluated.

In our study, *Acvr1b* and *Tgfbr1* were knocked out individually or in combination within myofibers. We showed that the expression levels of both genes were decreased. We have now quantified the phosphorylated Smad2/3 levels by western blotting in both TA muscles and EDL muscles.

Although p-Smad2/Smad2/3 and p-Smad3/Smad2/3 levels were not significantly different in TA muscles of Acvr1b^fl/fl^:Tgfbr1^fl/fl^ animals and controls, p-Smad2/Smad2/3 levels were reduced in EDL of Acvr1b^fl/fl^:Tgfbr1^fl/fl^ animals compared to control animals. In TA of Acvr1b^fl/fl^:Tgfbr1^fl/fl^ animals, p-Smad2/smad2/3 and p-Smad3/Smad2/3 relative intensity levels were 90 and 60% lower than those in controls, and in EDL this was 30 and 10% lower, respectively. The large variances in western blot band intensities made it difficult to conclude the effects of simultaneous TGF-β receptors knockout on Smad2/3 phosphorylation.

Note, however, that in the current study, *Acvr1b* and *Tgfbr1* genes were knocked out specifically within skeletal myofibres. Skeletal muscle contains more cell types than only myofibres, such as immune cells, fibroblasts and satellite cells, in which signalling via Smad2/3 is expected to be unaffected in our model. Based on H&E and IF staining, the number of mononucleated cells appeared to be substantially higher in TA of Acvr1b^fl/fl^:Tgfbr1^fl/fl^ animals (Figure 1 D, 2 C). This issue will be further addressed in the next paragraph. It is conceivable that Smad2/3 phosphorylation within other cells in the muscle will have masked in part the effect of simultaneous knockout of *Acvr1b* and *Tgfbr1* within the myofibre.

Effects of single knockout of either *Acvr1b* or *Tgfbr1* were more ambiguous, as in some cases Smad2/3 phosphorylation was higher than that in control. It may be due to the functional redundancy of these two receptors such that when only one receptor type was knocked out, ligands may signal via the other receptor to activate Smad2/3 signalling. Unchanged Smad2/3 phosphorylation is in line with the lack of phenotypical changes in Acvr1b^fl/fl^ and Tgfbr1^fl/fl^ animals compared to control animals.

Western blot results of p-Smad2/3 in TA and EDL are now presented.

We have added the following text:

Results section:

“Phosphorylation of TGF-β type I receptor is known to activate canonical Smad2/3 signalling. Therefore, we examined the effects of TGF-β type I receptor knockout on Smad2/3 phosphorylation in both TA and EDL muscle (Figure 3B). Single knockout did not affect phosphorylated / total protein ratios for Smad2 and Smad3 in muscles of Acvr1b^fl/fl^ or Tgfbr1^fl/fl^ mice, which was in line with the lack of effect on muscle size and phenotype and suggested that at least the presence of one of the two receptors was sufficient to maintain the Smad signalling. With regard to Smad2/3 phosphorylation in TA and EDL of Acvr1b^fl/fl:^Tgfbr1^fl/fl^ mice, a 31% reduction was shown for phosphorylation of Smad2 in EDL while phosphorylated levels of Smads2 and 3 tended to be reduced in TA.”

Discussion section:

“In addition, reduced Smad2/3 phosphorylation within myofibres likely contributes to the increase in Akt signalling and reduction in E3 ligase expression. In TA and EDL of Acvr1b^fl/fl^:Tgfbr1^fl/fl^ animals, Smad2/3 phosphorylation was or tended to be reduced, respectively. Note that TGF-β type I receptors were specifically knocked out in myofibres and that Smad2/3 in various other cell types can still be phosphorylated by TGF-β1, myostatin and activin A, masking the changes in myofibres.”

“In muscles that lack either *Acvr1b* or *Tgfbr1,* we observed no changes in Smad2/3 signalling, which is in accordance with the observation there is no or modest effect on muscle hypertrophy.”

Methods section:

“GM tissue and whole EDL muscles were lysed (Potter S 8533024, B. BRAUN) in RIPA buffer (Σ-Aldrich, R0278, Saint Louis, MO, USA) containing 1 tablet of protease inhibitor (Σ-Aldrich, 11836153001) and 1 tablet of phosStop (Σ-Aldrich, 04906837001) per 10ml.”

“1:500 for Phospho-Smad2 (Ser465/467) (138D4) (Rabbit mAb, 3108, Cell signaling, USA), 1:500 Phospho-Smad3 (Ser423/425) (C25A9) (Rabbit mAb, 9520, Cell signalling, USA), 1:500 for Purified mouse anti-Smad2/3 (610843, BD Biosciences, USA).”

Figures: Figure 3B has been added.

Lastly, the study needs to clearly identify the cell types affected by the myofiber-specific KO during regeneration induced by acute tissue damage (reviewer #2).

We agree that identification of the cell types will support the claim that enhanced myogenesis occurs within the double KO muscles and that the role of the immune cells and fibroblasts is enhanced in mouse muscle that lacks both *Acvr1b* and *Tgfbr1.* Such data will provide insight into the impact of the double knockout of the *Acvr1b* and *Tgfbr1*. Below we explain how we have added new data for the three different cell types as below.

1) Identification of proliferating and differentiating MuSCs:

To assess the total number of satellite cells and the number of proliferating MusCs and their state of activation, we performed staining for Ki67^+^/Pax7^+^/DAPI cells or Myogenin^+^/DAPI cells in TA muscle at day 0, 2 and 4 and quantified their numbers. In the original manuscript, we showed that at day 0 the number of satellite cells in Acvr1b^fl/fl^:Tgfbr1^fl/fl^ animals was already 3 times more than that of control muscles. The additional analyses now showed that at each time point the density of Ki67^+^/Pax7^+^ cells in the injured region was not different between control and Acvr1b^fl/fl^:Tgfbr1^fl/fl^ animals, indicating that the number of proliferating satellite cells was not affected by the knockout of the receptors. However, at day 4 post injury, we found more than 2-fold higher number of both Pax7^+^ and Myogenin^+^ cells, indicating that the combined knockout of both receptors enhanced myogenic differentiation of SCs in the Acvr1b^fl/fl^:Tgfbr1^fl/fl.^ Together these data suggest that in Acvr1b^fl/fl^:Tgfbr1^fl/fl^ animals regeneration in TA of is likely associated with an increased number of SCs already present within the muscle before the cardiotoxin-induced injury and that proliferation as well as SC differentiation were promoted before and upon cardiotoxin induced injury.

Data of dual staining for Ki67/Pax7/DAPI and Myogenin/DAPI cells are presented in the Results section and discussed. The following text and figures have been added:

Results section:

“On day 0, the number of proliferating cells (Ki67^+^) in low oxidative region of TA of Acvr1b^fl/fl^:Tgfbr1^fl/fl^ animals was about 7.6-fold higher than that in control animals (Figure 5E). Two days after injury, a 6-fold increase of proliferating cells was found in Acvr1b^fl/fl^:Tgfbr1^fl/fl^ animals compared to that on day 0. Moreover, at day 4 after injury, the number of proliferating cells in Acvr1b^fl/fl^:Tgfbr1^fl/fl^ animals was 1.7-fold higher than that in control animals. To determine whether the increased CSA of regenerating myofibres in Acvr1b^fl/fl^:Tgfbr1^fl/fl^ animals was due to an increased SCs number and advanced differentiation of myoblasts, we tested SCs proliferation and activation status. Although at day 0 and 4, the number of SCs (Pax7^+^) cells was more in Acvr1b^fl/fl^:Tgfbr1^fl/fl^ animals compared to that in control animals (Figure 5F), the number of proliferating SCs (Ki67^+^/Pax7^+^) did not differ from that in control (Figure 5G, Figure 5—figure supplement 2). Nevertheless, an accelerated rate of increase in Ki67^+^/Pax7^+^ cells was shown. Note that, at day 4 after injury the number of myogenin^+^ cells was more than 2.2-fold higher in Acvr1b^fl/fl^:Tgfbr1^fl/fl^ animals (Figure 5H, Figure 5—figure supplement 3). These findings indicate that muscle regeneration upon acute injury was improved in Acvr1b^fl/fl^:Tgfbr1^fl/fl^ animals, which was attributed to an accelerated myogenic process.”

Discussion section:

“Moreover, for Acvr1b^fl/fl^:Tgfbr1^fl/fl^ animals the numbers of SCs and differentiating myoblasts within the injured regions 4 days post injury were more than doubled compared to those in control animals. The observation that at all time points the number of proliferating SCs (i.e. Ki67^+^/Pax7^+^ cells) was not different between control and Acvr1b^fl/fl^:Tgfbr1^fl/fl^ animals indicates that in the Acvr1b^fl/fl^:Tgfbr1^fl/fl^ animals muscle damage had initiated activation and proliferation in the injured region between day 2 and 4. Alternatively, SCs had migrated from adjacent myofibres to the site of injury or from intact regions along the myofibres [3, 4]. The accelerated myoblast proliferation and differentiation likely contributed to the enhanced protein synthesis and hypertrophy of newly formed myofibres.”

Methods section:

“The densities of Ki67^+^, Pax7^+^, Ki67^+^/Pax7^+^ and Myogenin^+^ cells in TA were determined by counting the number of cells per mm^2^ muscle CSA. Ten images in low oxidative region of TA on day 0, 2 or 4 were randomly selected. All analyses were performed at 20× magnification.”

Figures: Figures 5 E, F, G and H and figure 5—figure supplement 1, 2, 3 have been added.

2) Identification of immune cells:

In the original manuscript, we had shown that at day 0 in TA muscles of Acvr1b^fl/fl^:Tgfbr1^fl/fl^ animals, *Tgf-β*, *Cd68* and *Cd163* mRNA expression levels were enhanced compared to those of control animals (Figure 4 D). In order to characterise the mononucleated cells that infiltrated the muscles of Acvr1b^fl/fl^:Tgfbr1^fl/fl^ animals at day 0, we now have performed immunofluorescent stainings to identify F4/80^+^ macrophages. These results are now presented in figure 2C. In line with the gene expression levels of macrophage markers, we now also show that prior to cardiotoxin-induced injury, the macrophage number in TA muscle of Acvr1b^fl/fl^:Tgfbr1^fl/fl^ animals was already 14-fold higher than that in control muscles.

To determine the effect of in Acvr1b and Tgfbr1 knockout on the number of macrophages during regeneration after muscle injury, we now present macrophage numbers within the injured region in figure 5C and figure 5—figure supplement 1. At day 4, more F4/80^+^ macrophages were found in the injured than that of control muscles. Macrophages play an important role in muscle regeneration as anti-inflammatory macrophages have been reported to stimulate myogenic cell differentiation while depletion of F4/80 macrophages attenuates the radial growth of myotubes and myofibers within the injured region [5]. Moreover, M2 macrophages have been shown to be pivotal for myofibre regeneration and maintaining myofibre size [6]. The observed increased macrophage gene expressions and higher macrophage cell number at day 0 and day 4 in the current study indicate an enhanced immune response by simultaneous knockout of Acvr1b and Tgfbr1 which likely have facilitated muscle regeneration.

Data of staining for F4/80 at day 0, 2 and 4 are now presented in the Results section and discussed. The following text and figures have been added:

Results section:

“We next characterised cells surrounding the spontaneously regenerating regions in TA as being macrophages or fibroblasts. F4/80 staining showed that the number of macrophages per mm^2^ muscle CSA in Acvr1b^fl/fl^:Tgfbr1^fl/fl^ animals was increased by 14-fold (23.7 cells/mm^2^) compared to that in control (1.7 cells/mm^2^) and Acvr1b^fl/fl^ animals (1.7 cells/mm^2^), while in Tgfbr1^fl/fl^ animals (4.5 cells/mm^2^) the number of macrophages per mm^2^ did not differ compared to that in the other three groups (Figure 2C).”

“Next, we hypothesized an increased immune response was involved in the accelerated muscle regeneration process after cardiotoxin induced muscle injury in the absence of Acvr1b and Tgfbr1. Macrophages were identified by F4/80 IF staining (Figure 5—figure supplement 1). The number of macrophages in TA of Acvr1b^fl/fl^:Tgfbr1^fl/fl^ animals at day 4 post injury was significantly increased compared to that in control muscle (Figure 5C).”

Discussion section:

“Macrophages play an important role in regulation of muscle regeneration [7]. Macrophages are classified in M1 (pro-inflammatory) and M2 (anti-inflammatory) macrophages [8]. Early after injury, gene expression of pan-macrophages marker *Cd68* and M2 macrophage marker *Cd163* [9], as well as the number of macrophages were increased in both control and Acvr1b^fl/fl^:Tgfbr1^fl/fl^ animals. Expression levels of *Il-6* and *Il-1β*, which are typical cytokines expressed by M1 macrophages, were not higher than in control animals, while *Igf-1ea* expression, also known to be expressed by M1 macrophages, was increased in Acvr1b^fl/fl^:Tgfbr1^fl/fl^ animals which was likely advantageous to expand the SC pool and to induce hypertrophy of newly formed myofibres. Moreover, TGF-β1 expression was increased in muscle with simultaneous knockout of Acvr1b^fl/fl^:Tgfbr1^fl/fl^. At a later stage after injury, M2 macrophages are known to promote myogenic differentiation and stimulate ECM deposition by releasing TGF-β1 [5, 10]. Taken together, we conclude that muscle-specific lack of both receptors promotes an inflammatory response by enhanced infiltration of macrophages which is associated with accelerated muscle regeneration.”

Methods section:

“Within muscle sections stained for F4/80, the density of macrophages in TA at day 0 was determined in 10 randomly selected locations and at in least 3 locations within the injured region at day 2 and 4 after injury.”

Figures: Figure 2C, 5C and Figure 5—figure supplement 1 have been added.

3) Identification of fibroblasts

To explain why ECM gene expression was enhanced in muscles of Acvr1b^fl/fl^:Tgfbr1^fl/fl^ animals, we assessed whether there was an increase in the number of fibroblasts (using *Tcf4* and *Pdgfra*, as markers for fibroblasts/mesenchymal cells). We performed immunofluorescent stainings for TCF4 and PDGFRa. However, these stainings were unreliable due to the non-specific binding of antibodies. As an alternative solution to estimate fibroblast presence, we analysed *Tcf4* and *Pdgfra* mRNA expression levels using qPCR. The results were added to figure 6 C. At day 0, expression of *Tcf4* was solely enhanced in Acvr1b^fl/fl^ animals compared to control animals. At day 4 after induction of injury, *Tcf4* mRNA levels in Acvr1b^fl/fl^:Tgfbr1^fl/fl^ animals were enhanced compared to those in the other groups. *Pdgfra* mRNA expression levels at both day 0 and day 4 were significantly higher in TA of Acvr1b^fl/fl^:Tgfbr1^fl/fl^ animals than those in controls. These findings indicate that combined knockout of Acvr1b and Tgfbr1 in skeletal muscle increases infiltration of fibroblasts acutely after injury.

Based on our results it is not possible to draw a firm conclusion whether the number of fibroblasts within the muscle tissue of Acvr1b^fl/fl^:Tgfbr1^fl/fl^ animals was increased or not, although we consider this plausible. Nevertheless, regardless of the number of fibroblasts within the muscle tissue of Acvr1b^fl/fl^:Tgfbr1^fl/fl^ animals, the increased *Tgf-β1* expression levels expressed by the increased number of macrophages, was likely the stimulus for the enhanced activation of available fibroblasts, myoblasts, myotubes and to stimulate *Ctgf, Col1a1* and *Col3a1* mRNA expressions [11, 12]. We believe these data provide a plausible explanation for the enhanced ECM gene expression observed at day 0. In addition, upon injury, ECM gene expression levels were increased in all groups, which is in line with the observations by other studies showing that collagen expression is transiently increased during regeneration.

Gene expression levels of *Tcf4* and *Pdgfrα* at day 0, 2 and 4 have now been presented in the Results section and are discussed. The following text and figures have been added:

Results section:

“To determine whether the number of fibroblasts was increased in TA muscles by knockout of *Acvr1b* and *Tgfbr1*, we assessed relative mRNA expression levels of the fibroblast markers, transcription factor 4 (*Tcf4*) and platelet-derived growth factor receptor A (*Pdgfra*) [13]. At day 0, *Tcf4* expression levels of TA in Acvr1b^fl/fl^ mice were increased compared to those in control animals, but was not different from those in Tgfbr1^fl/fl^ or Acvr1b^fl/fl^:Tgfbr1^fl/fl^ animals. In Acvr1b^fl/fl^:Tgfbr1^fl/fl^ animals, relative *Pdgfra* expression levels were increased compared to those of control and Acvr1b^fl/fl^ animals. Noteworthy, four days post injury, both *Tcf4* and *Pdgfra* mRNA levels were increased in Acvr1b^fl/fl^:Tgfbr1^fl/fl^ animal compared to those in control mice (Figure 6C).”

Discussion section:

“This hypothesis is supported by the infiltration of fibroblasts in TA muscle of Acvr1b^fl/fl^:Tgfbr1^fl/fl^ animals in the absence of injury, as well as by increased gene expression levels of *Pdgfrα,* which is expressed in most fibroblasts subtypes [14]. Furthermore, after injury in Acvr1b^fl/fl^:Tgfbr1^fl/fl^ animals, *Tgfbr1, Tcf4* and *Pdgfrα* mRNA levels were increased, which indicates increased infiltration of fibroblasts upon injury.”

Figure: Figure 6 C

References:

1. van Dijk, M., et al., Reduced dietary intake of micronutrients with antioxidant properties negatively impacts muscle health in aged mice. J Cachexia Sarcopenia Muscle, 2018. 9(1): p. 146-159.

2. Smith, L.R. and E.R. Barton, SMASH – semi-automatic muscle analysis using segmentation of histology: a MATLAB application. Skelet Muscle, 2014. 4: p. 21.

3. Schultz, E., D.L. Jaryszak, and C.R. Valliere, Response of satellite cells to focal skeletal muscle injury. Muscle Nerve, 1985. 8(3): p. 217-22.

4. Ishido, M. and N. Kasuga, In situ real-time imaging of the satellite cells in rat intact and injured soleus muscles using quantum dots. Histochem Cell Biol, 2011. 135(1): p. 21-6.

5. Arnold, L., et al., Inflammatory monocytes recruited after skeletal muscle injury switch into antiinflammatory macrophages to support myogenesis. J Exp Med, 2007. 204(5): p. 1057-69.

6. Ruffell, D., et al., A CREB-C/EBPbeta cascade induces M2 macrophage-specific gene expression and promotes muscle injury repair. Proc Natl Acad Sci U S A, 2009. 106(41): p. 17475-80.

7. Tidball, J.G., Regulation of muscle growth and regeneration by the immune system. Nat Rev Immunol, 2017. 17(3): p. 165-178.

8. Mosser, D.M. and J.P. Edwards, Exploring the full spectrum of macrophage activation. Nat Rev Immunol, 2008. 8(12): p. 958-69.

9. Hu, J.M., et al., CD163 as a marker of M2 macrophage, contribute to predicte aggressiveness and prognosis of Kazakh esophageal squamous cell carcinoma. Oncotarget, 2017. 8(13): p. 21526-21538.

10. Novak, M.L., E.M. Weinheimer-Haus, and T.J. Koh, Macrophage activation and skeletal muscle healing following traumatic injury. J Pathol, 2014. 232(3): p. 344-55.

11. Ireland, L.V. and A. Mielgo, Macrophages and Fibroblasts, Key Players in Cancer Chemoresistance. Front Cell Dev Biol, 2018. 6: p. 131.

12. Shi, A., et al., Synergistic short-term and long-term effects of TGF-beta1 and 3 on collagen production in differentiating myoblasts. Biochem Biophys Res Commun, 2021. 547: p. 176-182.

13. Mathew, S.J., et al., Connective tissue fibroblasts and Tcf4 regulate myogenesis. Development, 2011. 138(2): p. 371-84.

14. Muhl, L., et al., Publisher Correction: Single-cell analysis uncovers fibroblast heterogeneity and criteria for fibroblast and mural cell identification and discrimination. Nat Commun, 2020. 11(1): p. 4493.

15. Mathieson, T., et al., Systematic analysis of protein turnover in primary cells. Nat Commun, 2018. 9(1): p. 689.

16. Jahn, H.M., et al., Refined protocols of tamoxifen injection for inducible DNA recombination in mouse astroglia. Sci Rep, 2018. 8(1): p. 5913.

17. Sohal, D.S., et al., Temporally regulated and tissue-specific gene manipulations in the adult and embryonic heart using a tamoxifen-inducible Cre protein. Circ Res, 2001. 89(1): p. 20-5.

18. van der Zwaard, S., et al., Maximal oxygen uptake is proportional to muscle fiber oxidative capacity, from chronic heart failure patients to professional cyclists. J Appl Physiol (1985), 2016. **121**(3): p. 636-45.

19. van der Laarse, W.J., P.C. Diegenbach, and G. Elzinga, Maximum rate of oxygen consumption and quantitative histochemistry of succinate dehydrogenase in single muscle fibres of *Xenopus* laevis. J Muscle Res Cell Motil, 1989. **10**(3): p. 221-8.

20. Des Tombe, A.L., et al., Calibrated histochemistry applied to oxygen supply and demand in hypertrophied rat myocardium. Microsc Res Tech, 2002. **58**(5): p. 412-20.

21. van Wessel, T., et al., The muscle fiber type-fiber size paradox: hypertrophy or oxidative metabolism? Eur J Appl Physiol, 2010. **110**(4): p. 665-94.

22. van der laarse, W., et al., Size Principle of Striated Muscle Cells. Netherlands Journal of Zoology, 1997. **48**: p. 213-223.

23. Schmalbruch, H. and U. Hellhammer, The number of nuclei in adult rat muscles with special reference to satellite cells. Anat Rec, 1977. **189**(2): p. 169-75.

24. Glass, D.J., Skeletal muscle hypertrophy and atrophy signaling pathways. Int J Biochem Cell Biol, 2005. **37**(10): p. 1974-84.

25. Hardy, D., et al., Comparative Study of Injury Models for Studying Muscle Regeneration in Mice. PLoS One, 2016. **11**(1): p. e0147198.
